# Rehabilitation among individuals with traumatic brain injury who intersect with the criminal justice system: A scoping review

**DOI:** 10.3389/fneur.2022.1052294

**Published:** 2023-01-17

**Authors:** Vincy Chan, Maria Jennifer Estrella, Shazray Syed, Allison Lopez, Riya Shah, Zoe Colclough, Jessica Babineau, Zacharie Beaulieu-Dearman, Angela Colantonio

**Affiliations:** ^1^KITE-Toronto Rehabilitation Institute, University Health Network, Toronto, ON, Canada; ^2^Institute of Health Policy, Management and Evaluation, University of Toronto, Toronto, ON, Canada; ^3^Rehabilitation Sciences Institute, University of Toronto, Toronto, ON, Canada; ^4^Department of Occupational Science and Occupational Therapy, University of Toronto, Toronto, ON, Canada; ^5^Department of Biology, University of Toronto, Mississauga, ON, Canada; ^6^Department of Forensic Science, University of Toronto, Mississauga, ON, Canada; ^7^Library and Information Services, University Health Network, Toronto, ON, Canada; ^8^The Institute for Education Research, University Health Network, Toronto, ON, Canada; ^9^Department of Health Sciences, Lakehead University, Thunder Bay, ON, Canada; ^10^Dalla Lana School of Public Health, University of Toronto, Toronto, ON, Canada

**Keywords:** criminal justice, rehabilitation, traumatic brain injury, knowledge synthesis, concussion

## Abstract

Traumatic brain injury (TBI), a leading cause of morbidity and mortality globally, is highly prevalent among individuals who intersect with the criminal justice system (CJS). It is well-established that TBI negatively impacts individuals' interactions both within the CJS and upon release and is associated with serious disciplinary charges and higher recidivism rates. Although rehabilitation is fundamental to TBI recovery, it is not known to what extent rehabilitation is available to, or used by, individuals who intersect with the CJS. This scoping review explores the availability and extent of rehabilitation for individuals with TBI who intersect with the CJS, based on available literature. A systematic search of electronic databases (MEDLINE, Embase, Cochrane CENTRAL Register of Clinical Trials, CINAHL, APA PsycINFO, Applied Social Sciences Index and Abstracts, and Proquest Nursing and Allied Health), relevant organizations' websites, and reference lists of eligible articles identified 22 peer-reviewed articles and 2 gray literature reports that met predetermined eligibility criteria. Extracted data were synthesized through a descriptive numerical summary and qualitative content analysis. This review provides evidence that existing rehabilitation interventions are already serving individuals with TBI with a history of CJS involvement; however, they rarely consider or acknowledge TBI or CJS in their interventions. Findings also suggest opportunities to integrate rehabilitation for individuals with TBI who intersect with the CJS through TBI screening, education on TBI within CJS settings, and linkages to the community to facilitate continuity of care. This review also highlights significant gaps in knowledge regarding sex, gender, and other intersecting factors. Research to understand how these experiences impact the rehabilitation process throughout the CJS is urgently needed to enable timely and appropriate rehabilitation and continuity of care for diverse individuals with TBI who intersect with the CJS.

## 1. Introduction

Traumatic brain injury (TBI) is a significant cause of mortality and disability globally and across ages (1, 2). Among individuals who intersect with or are involved in the criminal justice system (CJS), TBI is highly prevalent (3). Two meta-analyses reported a lifetime prevalence of TBI of 51% (4) and 60% (5) among people who experienced incarceration. Systematic reviews also reported prevalence rates of TBI of up to 100% across all ages (6), 72.1% among youths (7), and 85% among incarcerated adults (8). One study focusing on community corrections found a 47% prevalence rate of TBI among persons on probation (9). Studies that investigated sex-specific rates of TBI reported a 49% prevalence rate among female youths (10), 95% among female adult prisoners (10), and 63.7% among male adult prisoners (11). These prevalence rates far exceed those reported for the general population (2.0–38.5%) (4).

Broadly, TBI is associated with cognitive (12, 13), social, and psychological sequelae, including problematic substance use (13). These challenges may influence how an individual behaves and concurrently, how their behavior is perceived. For example, cognitive sequelae, such as memory and attention deficits may result in an individual not remembering rules, not responding, or being slow to respond to directions (14); forgetting an appointment, event, or conversation (15); or having difficulty articulating their thoughts and comprehending court and criminal proceedings (16). These behaviors may be misconstrued and viewed as defiant or uncooperative, leading to disciplinary actions by CJS staff (14). This risk is substantiated by research showing that incarcerated individuals who have a history of TBI are more likely to incur serious disciplinary charges (17) and behavioral infractions (e.g., refusing orders, possessing drugs and weapons, attempting to escape, or failing to make a required appearance) and have higher recidivism rates compared to those without TBI (5, 18).

Rehabilitation or interventions that aim to reduce disability and enhance functioning among individuals in interaction with their environment (19) is fundamental to recovery from TBI (19–21). TBI rehabilitation, which encompasses assessment and management of TBI sequelae, is critical to addressing TBI symptoms, improving functional status, and facilitating return-to-work (22, 23) and community integration or reintegration (24, 25). However, most reviews on TBI within the CJS have focused on TBI prevalence and are limited to the corrections setting (4–8, 10, 26). As such, there is a significant knowledge gap regarding the availability of rehabilitation both in the corrections setting as well as in other parts of the CJS (e.g., policing, court, and parole) (27–29).

This scoping review addresses these knowledge gaps by exploring the types of rehabilitation interventions available to, or used by, individuals with TBI who intersect with all parts of the CJS—i.e., involvement with policing (i.e., police interactions and arrests), courts (i.e., trials, including prosecution, adjudication, and sentencing), corrections (i.e., detention), and parole (i.e., parole and probation) (27–29). Overall, this review provides a comprehensive summary of rehabilitation based on available literature for individuals with TBI who intersect with the CJS and discusses (1) opportunities to integrate rehabilitation for this population and (2) future research directions.

## 2. Methods and analysis

The protocol for this scoping review is published in a peer-reviewed journal (30) and is summarized below. It was developed using methodology frameworks from Levac and colleagues (31) and Arksey and O'Malley (32). The reporting of this scoping review followed the Preferred Reporting Items for Systematic Reviews and Meta-Analyses extension for Scoping Reviews (PRISMA-ScR) (33), and the search strategy is reported in accordance with the PRISMA-S extension (34).

### 2.1. Identifying the research question

This scoping review answered the research question “what are the types of rehabilitation interventions and/or programs available to, or used by, individuals with TBI who intersect with the CJS?” Table 1 defines the concepts of TBI, CJS, and rehabilitation and are used to guide the search strategy, study selection process, charting of the data, and reporting of the findings.

**Table 1:** Definitions for TBI, CJS, and rehabilitation.

**Concept**	**Definition**
Traumatic brain injury (TBI)	“An alteration in brain function, or other evidence of brain pathology, caused by an external force” (35)
Criminal justice system (CJS)	Interaction with the CJS may include any of the following stages, described in the CJS for Canada (27), United Kingdom (28) and United States (29): • Policing: Involvement with police interactions and arrests • Courts: Involvement with trials, including prosecution, adjudication, and sentencing • Corrections: Involvement in the detention stage of the criminal justice system • Parole: Involvement in parole and probation stage
Rehabilitation	“A set of interventions designed to optimize functioning and reduce disability in individuals with health conditions in interaction with their environment” (19) or healthcare providers/professional disciplines identified in clinical practice guidelines for rehabilitation for TBI (36, 37): • Neuropsychologist and psychometrist • Nurse • Nutritionist • Occupational therapist • Physician and/or physiatrist • Physiotherapist • Psychologist with expertise in behavioral therapy • Rehabilitation support personnel • Social worker • Speech-language pathologists • Therapeutic recreationist

### 2.2. Identifying relevant studies

The search strategy for this scoping review was informed by previous scoping and systematic reviews (38–40) and developed in collaboration with an Information Specialist (JB) and team members with research and content expertise in TBI, CJS, and rehabilitation (VC, MJE, AC). The strategy for MEDLINE^®^ ALL (in Ovid, including Epub Ahead of Print, In-Process & Other Non-Indexed Citations, Ovid MEDLINE(R) Daily) database was first developed and subsequently translated to: Embase and Embase Classic (Ovid), Cochrane CENTRAL Register of Clinical Trials (Ovid), CINAHL (EBSCO), APA PsycINFO (Ovid), Applied Social Sciences Index and Abstracts (Proquest), Criminal Justice Abstracts (EBSCO), and Nursing and Allied Health (Proquest). Three concepts were used to develop and form the final search structure: (CJS) + (rehabilitation) + (TBI or cognitive impairment). This search strategy was used to identify peer-reviewed primary research and review articles. No date or language limits were placed on the search strategies and a filter to exclude animal studies was included (41). Searches were conducted in July 2021.

Gray literature, defined as reports from brain injury, CJS, and rehabilitation organizations, were identified from organizations' websites by manually searching the websites and through consultation with stakeholders (see Consultation section). Reference lists of included articles, gray literature reports, and scoping and systematic reviews that met the inclusion and exclusion criteria were searched for additional relevant literature using the criteria outlined below. Supplementary material 1 presents the search strategy for each database and the organizations that were searched for gray literature. EndNote X8.2 (42) was used for reference management and Covidence (43) was used for de-duplication and study selection.

### 2.3. Study selection

The following inclusion criteria was applied to all peer-reviewed articles and gray literature reports retrieved from the search strategy:

1. Describe or document (a) rehabilitation programs or interventions or (b) services provided by healthcare providers or professional disciplines, as defined in Table 1, and
2. Include individuals (of any proportion) with TBI, and
3. Include individuals (of any proportion) who intersected with any part of the CJS, as defined in Table 1, and
4. Report primary research findings.

The following were excluded from this review:

1. Books and conference proceedings, or
2. Articles, gray literature, and reviews that are narrative, commentaries, or describe a theory or framework without reporting primary research findings, or
3. Articles that describe a sample including individuals with brain injury or individuals experiencing cognitive impairment without specific mention of TBI.

All title and abstracts were independently screened in Covidence by two reviewers (RS, ZB) based on the above criteria. At this stage, articles that (a) included individuals with brain injury or individuals experiencing cognitive impairment without specific mention of TBI or (b) were scoping or systematic reviews that met the above criteria were also considered for full-text review. For all non-English language articles, the published English abstract was used to assess eligibility. A pilot screen of 20 title and abstracts was conducted until a minimum 80% agreement was reached between the two reviewers. The resulting agreement at the title and abstract screen was 95.7% (kappa 0.747).

At the full-text screen, three reviewers (SS, AL, ZC) participated in the screening of the articles, with each article independently screened by two reviewers. Primary research articles from the scoping and systematic literature reviews included at the title and abstract screen were extracted and screened based on the inclusion and exclusion criteria. All non-English language articles were translated to English using DeepL Translate (44) and/or Google Translate (45). A pilot screen of 10% of eligible full-text articles was conducted until a minimum 80% agreement was reached between the two reviewers. The resulting agreement at the full-text screen was 90.9% (kappa = 0.670). At both stages of the screening process, discrepancies between the two reviewers were resolved by consensus or consultation with a third reviewer (VC or MJE).

### 2.4. Charting the data

One reviewer independently charted the data (SS or AL), which were subsequently peer-reviewed by a second reviewer (SS or AL). A random sample of five articles were selected for charting until a minimum of 80% was reached between the two reviewers. Discrepancies in the charting of the data were resolved through consensus or review by a third reviewer (VC). Supplementary material 2 presents the charting table.

### 2.5. Collating, summarizing, and reporting the results

This stage of the scoping review was informed by the methodology framework described by Levac and colleagues (31). A descriptive numerical summary of the data presented in the charting table was conducted by three reviewers (VC, SS, AL) and qualitative content analytic techniques were applied by two reviewers (VC, MJE) to develop the categories presented below. The results from the quantitative summary and qualitative content analysis were used to apply meaning to the results, specifically in relation to our research question and in informing opportunities to integrate rehabilitation for individuals with TBI who intersect with the criminal justice system and future research directions.

Quality appraisal was conducted by one reviewer (VC) and peer-reviewed by a second reviewer (RS). The Study Quality Assessment Tools designed by the Research Triangle Institute International and the National Heart, Lung and Blood Institute of the National Institutes of Health (46) were used to inform the internal validity of the included articles. No articles were eliminated from this scoping review based on the results of the quality appraisal; findings were used to inform the process of applying meaning to the study. Supplementary material 3 presents the quality appraisal.

### 2.6. Consultation

Preliminary findings from this scoping review were presented to stakeholders including front-line staff and service providers in the CJS and brain injury sectors; health administrators, decision-makers, and policy-makers; health professionals who provide care for individuals with TBI and/or individuals who have intersected with the CJS; and researchers and trainees who conduct research on rehabilitation, TBI, and the CJS. These individuals form the Program Advisory Committee (PAC) of the Traumatic Brain injury in Underserved Populations Research Program (47, 48). Feedback received from the PAC meeting was recorded and integrated in this scoping review.

## 3. Results

**Figure 1:**
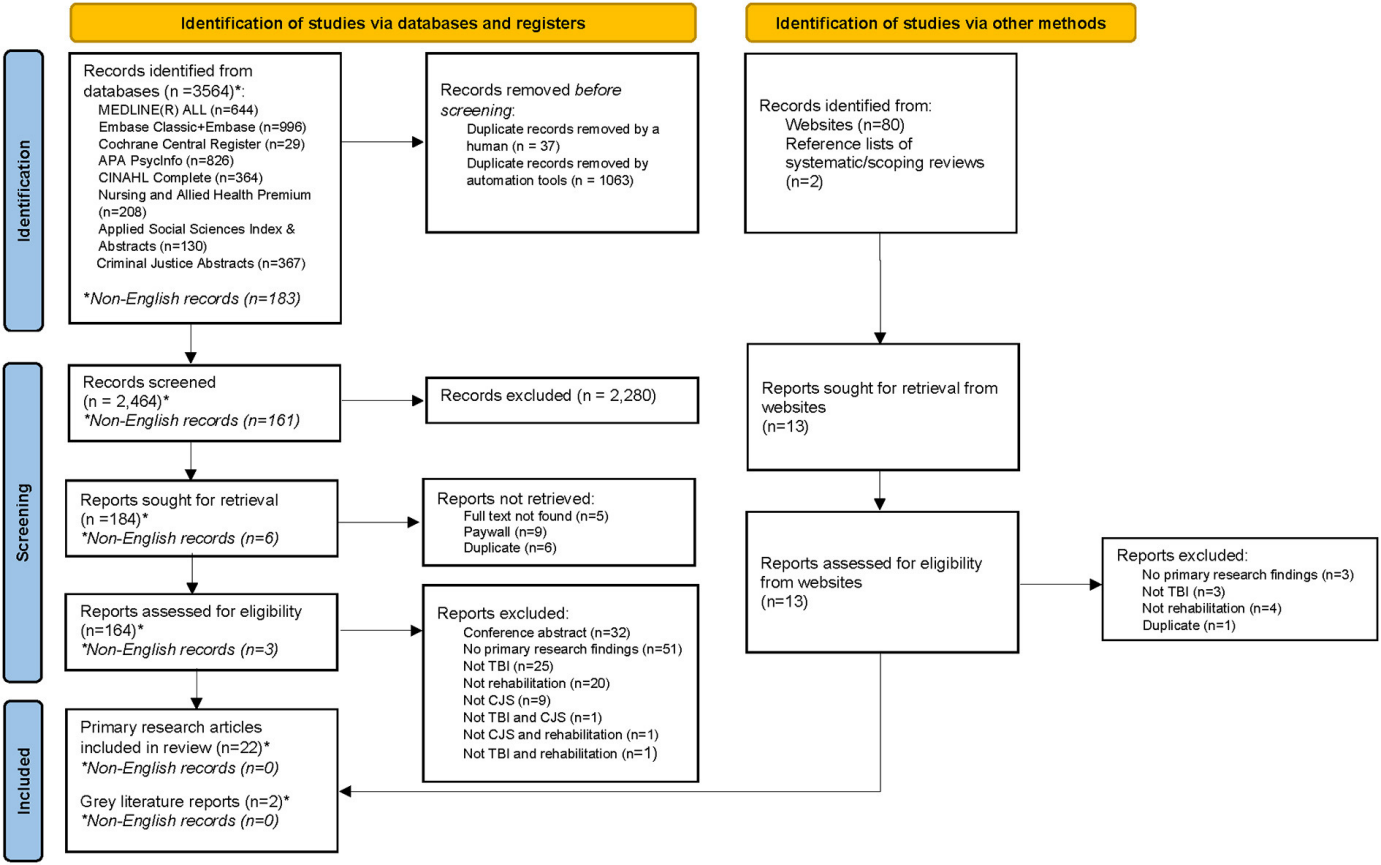
PRISMA flow diagram.

A total of 3,564 citations were identified from the search strategy for databases, of which 183 were in non-English language. Twenty-two primary research articles and two gray literature reports (all English language) met eligibility criteria and were included in the scoping review. Figure 1 presents the PRISMA Flow Chart describing the study selection process. The articles included in this review were published between 1991 and 2021. Eight articles (33.3%) described rehabilitation interventions specifically for individuals with TBI who intersect with the CJS (49–56), three of which were behavior-focused interventions (37.5%) (49–51) and the remainder (*N* = 5, 55.5%) described interventions that connected individuals to necessary supports to facilitate engagement, rehabilitation, and community re-integration (herein referred to as “linkage programs”) (52–56). Sixteen articles (66.7%) were health services-related articles that described the use of rehabilitation health services without specific information on the rehabilitation interventions (57–72). Table 2 presents the study characteristics, rehabilitation location(s), funding source of the rehabilitation intervention/programs, and CJS intersection(s) of the articles included in this review.

**Table 2:** Study characteristics.

**Characteristics**	***N* (%)**
**Country of study**	
United States (56, 57, 59, 61, 62, 64, 69, 70, 72)	9 (37.5)
United Kingdom (50, 52–55, 58, 60)	7 (29.2)
Canada (49, 68, 71)	3 (12.5)
Australia (63, 65)	2 (8.3)
New Zealand (51)	1 (4.2)
Poland (66)	1 (4.2)
Egypt, Honduras, Mexico, Palestine, and South Africa (67)	1 (4.2)
**Study design**	
Cohort (52–54, 56–58, 61, 63–65, 67, 69, 70, 72)	14 (58.3)
Case studies (49, 50, 55, 60, 66)	5 (20.8)
Randomized controlled trials (51, 68, 71)	3 (12.5)
Cross-sectional (59)	1 (4.2)
Before-after no control (62)	1 (4.2)
**Sex and gender** ^**a**^	
Males or men only (51–53, 56, 63, 65)	6 (25.0)
Females only (54)	1 (4.2)
**Rehabilitation locations**	
Secure settings (e.g., Jails or Prison) (51–54, 56)	6 (25.0)
Inpatient and outpatient rehabilitation (60, 61, 63, 65, 66, 69, 70, 72)	8 (33.3)
Residential rehabilitation (49, 57)	2 (8.3)
Community-based rehabilitation programs (50, 59, 64, 67, 68, 71)	6 (25.0)
Inpatient, outpatient, and secure settings (58)	1 (4.2)
**Funding source of rehabilitation program/interventions**	
Federally funded (56, 65, 69, 72)	4 (16.7)
Implemented by non-profit agency (52, 53)	2 (8.3)
Not reported (49–51, 54, 55, 57–64, 66–71)	18 (75.0)
**CJS intersection** ^**b**^	
Corrections (e.g., “arrested,” “incarcerated,” “in custody,” “convicted,” “in prison,” “in jail,” “forensic history,” “criminal charges”) (49–58, 60–65)	23 (95.8)
Parole/probation (54–57, 65, 68)	6 (25.0)
Courts (50, 55, 57, 59, 60)	5 (20.8)
Police (49, 59)	2 (8.3)

^a^Data excludes case studies; four studies reported examining gender (56, 59, 63, 65), however, three studies used the terms males or females to describe their sample (59, 63, 65); overall, the majority of participants were males or men, ranging from 56 to 100%.

^b^Ten articles described intersecting with more than one part of the CJS (41.7%) (49, 50, 54–57, 59, 60, 65, 68).

### 3.1. Rehabilitation interventions for individuals with TBI who intersect with the CJS (*N* = 8)

Eight articles described rehabilitation interventions that were developed or implemented specifically for individuals who experienced TBI and are involved with the CJS (49–56). These articles were further categorized based on the overarching goal(s) of the rehabilitation programs or interventions: (1) behavior-focused interventions (*N* = 3) (49–51) and (2) linkage programs (*N* = 5) (52–56).

#### 3.1.1. Behavior-focused Interventions (N = 3)

Three articles described rehabilitation interventions that aimed to improve coping strategies (51), manage sexually intrusive behavior (49), and address bullying (50) through behavioral (49) or cognitive-behavioral interventions, (49, 51) integrating mindfulness-based stress reduction (51) and building pro-social skills focused on anger management and moral development (50). Psychoeducation (49) and pharmacological interventions (49) were also considered, where appropriate. These interventions were delivered in individual (49) or group formats, (50, 51) which may include family members (49, 50) within a prison (51), psychiatric and residential treatment facility (49), and community-based neuro-behavioral rehabilitation unit (50). Psychologists (51), psychosocial counselors, education specialists, neuropsychiatric consultants (49), and community placement personnel (49) were involved in the interventions.

In all three articles, interventions were modified to account for TBI-related impairments. These included psychoeducation on TBI and its possible effects specific to the prison environment (49, 51); using prison-specific examples during the intervention/program (51) or behavioral examples (e.g., role playing) (50); and repeating materials from the previous session (49, 51) to promote recall and retention. Other adaptations for TBI were related to sessions' schedule and duration, consistency, and communication; for example, shortening the session time; scheduling sessions when clients were most alert; ensuring consistency in group facilitators; and using concrete and literal communication and avoiding humor (50). Information on TBI screening and/or diagnosis was not reported; only one article reported asking participants about possible lifetime TBIs. However, information on screening or interview questions was not provided (51). One article highlighted the need for a multidisciplinary approach in assessing and treating sexually intrusive behaviors associated with TBI (49).

#### 3.1.2. Linkage programs (N = 5)

Five articles described linkage programs or programs that connected individuals in prisons to necessary supports to facilitate engagement, rehabilitation, and community re-integration (54–56) and a juvenile custodial secure facility (52, 53). These programs were the “Brain Injury Linkworker Service” (52–55) or “NeuroResource Facilitation” (56). Four of the five articles described linkage programs for adults (52, 53, 55, 56), one of which focused specifically on females (54), and the remaining article focused on young adults (53).

A key component of the Brain Injury Linkworker Service is the Linkworker who connected with other staff and provided resources to participants. Linkworkers provided education on TBI and its effects on behavior to clients, healthcare workers, and CJS staff; connected with agencies and families outside of the prison to support transition and community re-integration; and delivered support sessions aimed at generalizing strategies learned during custody to the current home environment. The NeuroResource Facilitation program is similar to the Brain Injury Linkworker Services, such that a NeuroResource Facilitator identified resources and provided support to individuals and their families, including but not limited to brain injury education, advocacy, and development of compensatory strategies for successful community living (56). Two articles noted that the Linkworkers and the NeuroResource Facilitator were “psychology graduates” who were gaining experience prior to becoming professionally trained in clinical psychology or a related field (55) or a “brain injury specialist” with more than 20 years of experience working with individuals with brain injury (56). The remaining articles did not describe the Linkworkers' professional backgrounds. All linkage programs involved TBI screening and assessment, dedicated/one-to-one support in custody, and discharge planning and community interventions.

##### 3.1.2.1. TBI screening and assessment

Screening for TBI was conducted prior to referral to a linkage program using the Neurodisability section of the Comprehensive Health Assessment Tool (CHAT) (52, 53), the Brain Injury Screening Index (BISI) (53–55), or the Traumatic Brain Injury Questionnaire (TBIQ) (56). Three articles did not report who conducted the TBI screening while two articles noted nurses (52) or mental health nurses (53) completed the Neurodisability section of the CHAT. Those who screened positive for TBI were further assessed through neurocognitive tests, standardized assessments (53, 55, 56) and/or a review of medical records (52, 53, 56), clinical interviews (53–55), and liaison with family members and professionals (52, 53, 55). The neurocognitive tests focused on areas relevant to TBI such as memory and executive functioning and explored specific areas of need (52, 55) and identified cognitive impairments that could impact community re-entry (56). These assessments informed the development of goals, participants' opinion of their needs (52, 53, 73) or an individualized intervention plan (54) that guided the delivery of dedicated/one-to-one supports.

##### 3.1.2.2. Dedicated/one-to-one support in custody

While in custody, all interventions offered direct support for individuals that are tailored to their needs and are continuously reviewed as part of their custodial sentence plan. These direct supports included psychoeducation on TBI and its consequences (52–56) and strategies (e.g., developing an external structure or routine and assisting the individual to organize themselves and their tasks) and functional aids (e.g., thought records, diaries, and prompt cards) to cope with impairments associated with TBI (52, 53, 55, 56). Other TBI-focused supports included cognitive assessment and remediation and emotional management, behavioral management and health and well-being (e.g., sleep and epilepsy) (54); transportation training, medical case management (56); and advocacy (54, 56). Supports specific to the Brain Injury Linkworker Services for youth included the co-development of behavior support plans and supports for problem solving difficulties in the classroom setting, attending professional meetings, and appearing in court (53).

Aside from directly supporting the individual with TBI, Linkworkers and NeuroResource Facilitators also provided indirect support by providing brain injury education and awareness training to professionals who worked with them, for example education staff, mental health nurses, and key workers (52, 53), class leaders (52, 53, 56), prison (54, 55), probation, and health staff, and key officers (54). Specifically, Linkworkers provided education on the impact of TBI on the individual's behavior, its impact on the care they are receiving, and guidelines on how to best engage and support the individual so that the intervention accounts for the challenges they experience as a result of their TBI (53, 55). They also supported the development of behavioral or individualized intervention plans (52, 53) that were implemented by other support staff (54). Three articles reported the benefit of having the Linkworker be a part of multidisciplinary meetings to provide information regarding TBI and the individual so TBI can be considered into the care plan and to facilitate referral to other services (52–54).

##### 3.1.2.3. Discharge planning and community interventions

Interventions were adapted prior to release to include the development of a care or support plan and discharge summary; liaising with healthcare professionals, prison staff, and agencies; and ongoing individualized support, including connecting to necessary resources. Specifically, Linkworkers or NeuroResource Facilitators developed a support plan with the individual that identified goals, areas of concerns, and risks (56); provided education on their brain injury (56); developed strategies to manage TBI-related impairments (54); and ensured that a support system (i.e., resources, requirements, and supports) is in place (55, 56) and improvements that were made during custody are retained post-release (52, 53). A “discharge summary” (52), “discharge pack” (55), or “pre-release workbook” (54) was created and used to engage with the justice team and general practitioner (52, 53); the department of corrections and parole staff (56); and prison, probation, and health staff (54) to ensure that needs related to TBI are continuously supported in the community (52, 53). “Portable profiles” (55) of the individual's TBI history, its potential influence on behavior, and information on how to best support the individual were also shared with community agencies and families (55). Partnerships or relationships with community organizations were highlighted as essential during this phase (55, 56). For example, the contributions of the Department of Corrections and Board of Probation and Parole in the United States were key in identifying options for placement post-release and in allowing facilitators to provide hearing examiners with information about the individuals' TBI (56). Finally, ongoing individualized supports or “through the gate” supports (55) were provided to support TBI-related needs (52–54), monitor release plans (56), and aid in generalizing strategies learned in custody (55). Examples of ongoing individualized supports included meeting with individuals in their community placement or home and helping them pursue community programs and services [e.g., housing, education, employment and training opportunities, and brain injury services (52, 53, 56)].

### 3.2. Use of rehabilitation interventions by individuals who experienced a TBI and intersected with the CJS (N = 16)

Sixteen of twenty-four articles documented the use of rehabilitation interventions without specific details on the interventions. Articles in this category were further classified into two sub-categories: (1) rehabilitation interventions in the CJS setting or specifically for individuals who intersect with the CJS and (2) rehabilitation interventions that do not focus on the CJS but include individuals with a history of CJS involvement.

#### 3.2.1. Rehabilitation interventions in the CJS setting or for individuals who intersect with the CJS (N = 4)

Four of sixteen articles documented rehabilitation interventions specifically for CJS-involved individuals (57, 59) or interventions that were offered in the CJS setting (58, 60). In one article, all participants intersected with the CJS (57) and another documented a case study of an individual in prison (60); the remaining two articles documented 25.0% (58) and 31.8% (59) of CJS-involved individuals. The proportion of individuals who sustained a TBI ranged from 36 to 100%. Two of the four articles focused on individuals who committed law-violating behavior (e.g., substance use, theft, and physical assault) and received rehabilitation in neurorehabilitation centers (59, 60). One described a residential alcohol program for individuals driving under the influence (57), and the remaining article documented behavioral health, physical health, and criminal justice and social services offered in inpatient, outpatient, and jail and prison settings (58). None of the articles documented the health professionals involved in the rehabilitation interventions.

Two articles reported considerations or adaptations for TBI (57, 59), specifically in the form of TBI education. The residential alcohol program incorporated one classroom session to raise awareness of TBI and its interaction with alcohol (57). The community-based educational program for students with TBI integrated staff training and provided educational materials and support strategies for public school teachers in their program (74). Education and training on TBI centered on brain injury and its effect on learning development and psychological adjustment; teaching strategies to accommodate challenges common to students with TBI; communicating effectively with families; writing the individual educational plan; medication administration; and crisis prevention intervention. Examples of strategies included modifying the environment to prevent over-stimulation and using assistive devices to address deficits in memory. A multidisciplinary team of nursing services, education, family, community living, and staff consultants (e.g., speech and language pathology, occupational therapy, and physical therapy) formed the educational team and developed and implemented students' individualized educational plans.

#### 3.2.2. Rehabilitation interventions that do not focus on the CJS (N = 12)

Twelve articles documented rehabilitation interventions that did not focus on CJS-involved individuals or were not delivered in the CJS setting. However, the proportion of individuals with a history of CJS involvement ranged from 8.8 to 70.7% (excluding case studies) while the proportion of individuals with TBI ranged from 52.4 to 100%. Six of the twelve articles documented rehabilitation interventions specifically for individuals with TBI within an inpatient rehabilitation setting (63, 69, 70, 72) or outpatient rehabilitation medicine clinic (61, 65). These articles only documented that their sample included individuals with TBI who intersected with the CJS; no information was provided on the interventions and thus, it is not clear how or if interventions were tailored specifically to CJS-involved individuals.

The remaining six articles in this category documented rehabilitation interventions addressing specific needs [i.e., housing (68, 71), employment (62)] or specific sub-populations [i.e., individuals with co-occurring conditions (64, 66) and torture survivors (67)]. Rehabilitation interventions included Housing First, where individuals received either assertive community treatment, intensive case management, or treatment as usual depending on level of need (68) or through randomization (71); supported employment (62); comprehensive services for torture survivors through torture rehabilitation centers (67); community supports for individuals with co-occurring conditions (64); and psychotherapy and neuropsychological rehabilitation for an individual with schizophrenia and TBI (66). Only one article documented rehabilitation professionals involved in supporting torture survivors (i.e., service providers from medicine, psychology, nursing, and social work) (67). Two of these six articles reported considerations for TBI (64, 66), but none reported considerations for the CJS setting or CJS-involved individuals. The neuropsychological program described by Pachalska and colleagues (66) targeted working memory, perseveration, neglect, and executive functions and utilized elements of cognitive therapy and art therapy. The intervention began with neuropsychological tests followed by specific therapeutic tasks that aimed to address TBI-related impairments (66). Ylvisaker and colleagues described an intervention that addressed challenging behaviors associated with TBI, where screening and assessment of behavior support needs were incorporated in the intervention (64).

## 4. Discussion

This scoping review explored the extent to which rehabilitation, including the types of rehabilitation programs or interventions, is available to, or used by, individuals with TBI who intersected with the CJS. A systematic search of the literature identified 22 primary research articles and 2 gray literature reports describing programs/interventions specifically for individuals with TBI who intersect with the CJS (49–56) or health services-related articles that described the use of rehabilitation health services without specific information on the rehabilitation intervention (57–72). Only 8 articles reported health professionals who contributed to the rehabilitation programs/interventions (51–56, 67, 70). The majority of the articles described rehabilitation health services used within inpatient and/or outpatient rehabilitation centers (60, 61, 63, 65, 66, 69, 70, 72), followed by rehabilitation programs offered within secure settings (51–54, 56), rehabilitation interventions within the community (50, 59, 64, 67, 68, 71), and residential rehabilitation (49, 57). Below, we discuss key findings in relation to (a) opportunities to integrate rehabilitation for individuals with TBI who intersect with the CJS and (b) recommendations for future research.

### 4.1. Opportunities to integrate rehabilitation

This scoping review provides evidence that existing rehabilitation programs/interventions that are not specifically developed for individuals with TBI who intersect with the CJS are already serving these individuals. Of the four rehabilitation interventions provided within the CJS setting or for individuals who intersect with the CJS (57–60), between 36 and 100% of participants experienced a TBI, however, only two articles reported considerations or adaptations for TBI (57, 59). Similarly, of the 12 articles that described use of rehabilitation interventions, only the proportion of individuals who intersected with the CJS (up to 72.7%) and the proportion of individuals with a history of TBI (up to 100%) were reported. Furthermore, only two articles reported considerations for TBI (64, 66) and none of the articles reported considerations for CJS history. These findings suggest missed opportunities to integrate rehabilitation for individuals with TBI who intersect with the CJS through (1) TBI screening and (2) education embedded within CJS settings, and (3) linkages to the community to facilitate continuity of care.

First, the findings from this review confirmed the need for TBI screening as a critical first step in facilitating access to appropriate intervention to individuals who intersect with the CJS. Specifically, TBI screening and additional assessments (e.g., neurocognitive tests and standardized assessments) helped identify unmet health needs (54) and supported the development of individualized intervention plans (52–54). Such intervention plans included psychoeducation on TBI and its consequences; strategies and functional aids to address TBI-related impairments; and modifications to interventions. The need for screening is not new and has been highlighted in studies that focused on underserved populations with TBI (40, 75–79). Unfortunately, despite literature supporting the need for TBI screening, almost half of the articles identified in this review did not specify how TBI was ascertained. Additionally, among the few articles that described TBI screening, only one article reported on considerations for screening, particularly for women in prison (54). This article described the need for a gender-informed screening process and the potential implications of screening (i.e., negative treatment from staff and other individuals in prison and unmet expectations regarding supports after screening) among women in prison. No further information on barriers and facilitators to TBI screening in different parts of the CJS were reported apart from these considerations. The limited information on screening in the CJS context makes it challenging to integrate routine TBI screening in all parts of the CJS, leading to missed opportunities for individuals with TBI to access appropriate interventions (79). More broadly, TBI screening could also result in more accurate estimates of the prevalence of TBI in the CJS context (79) and to support the development of new interventions, further research, and educational opportunities for health professionals working with this group (79). As such, research on the barriers and facilitators to screening for TBI among individuals who intersect with the CJS is urgently needed to inform the feasibility, processes and implications of TBI screening for individuals with lived experience.

A second opportunity to integrate rehabilitation in the CJS context is to educate individuals with lived experience of TBI, healthcare professionals, and CJS staff (e.g., parole officers, correctional officers). In the articles included in this review, education encompassed the impact of TBI on behavior in relation to a particular context (e.g., in prison, in the classroom among juvenile offenders) (49, 51–54, 64), other consequences of TBI (e.g., interactions with substances) (57), strategies to manage TBI-related impairments for individuals with TBI (52–56), and for healthcare professionals and staff, guidelines on engaging and supporting individuals with TBI (52–56). The lack of training among service providers supporting individuals with TBI has been highlighted extensively in the literature, particularly among underserved populations (75–77), service users and families interacting with community services (80), in the context of TBI and mental health/substance use (81), and in some parts of the CJS (82, 83). These studies also highlighted the importance of education in increasing TBI awareness and knowledge regarding appropriate supports and addressing negative views about TBI (82, 84). Specific to the prison setting, educating staff on TBI and TBI-related behaviors and strategies to manage these behaviors could lead to decreased penalties and reduced negative interactions between staff and individuals with TBI in prison (85). Unfortunately, similar to TBI screening, none of the articles that documented education described specific approaches, barriers, or facilitators, which makes it challenging to implement TBI education in the CJS context. Additionally, education was often limited to a small sample of individuals in corrections, residential programs, or frontline staff in the community; as such, information regarding the feasibility of educating individuals and staff in other parts of the CJS (e.g., court, parole, etc.) is lacking. Research that explores the perspectives of healthcare professionals and CJS staff supporting this group is urgently needed to determine the feasibility of implementing education on TBI in all parts of the CJS. For example, opportunities for education may be restricted by privacy and access to certain settings within the CJS as well as willingness to participate in education programs among CJS staff. As such, research with individuals with lived experience of TBI and CJS involvement, healthcare professionals, and CJS staff to co-develop education materials that are appropriate and sensitive to the needs of these individuals are encouraged to understand when and how to initiate education regarding TBI.

Finally, this scoping review highlights an opportunity to address the current fragmented care for individuals with TBI (80, 84, 86–88), including the lack of continuity in resources and support in community re-integration, (80, 87, 88) through Linkworker or NeuroResource Facilitator roles. These individuals provided direct support to the client while in custody and indirectly supported them by engaging health professionals, prison staff that interact with the client (52–56). Upon release, they also collaborated with community organizations (e.g., Brain Injury organizations, organizations providing vocational rehabilitation) (52–56) and established partnerships with government agencies to ensure that needs related to TBI are continuously supported in the community (55, 56). Findings from the articles on the Brain Injury Linkworker Service and the NeuroResource Facilitation Program documented that the supports that are part of the linkage programs improved TBI-related impairments (e.g., memory, aggression, and mood) (52, 54); supported employment (52, 55), positive relationships (52), independent (55) and/or safe living (54), and facilitated access to rehabilitation (55) and health insurance (56). In addition to these outcomes, the integration of Linkworker or NeuroResource Facilitator into multidisciplinary team meetings has been noted to facilitate comprehensive assessments and connections to other services (52–54). It is noteworthy that the benefit of multidisciplinary teams has also been identified in research with individuals with TBI (89, 90) or underserved populations, specifically in tailoring interventions to accommodate for TBI impairments (75, 76, 91). As such, opportunities to integrate linkage programs or services within the broader CJS context should be explored, particularly those that address the noted barriers regarding the cost and resources required to implement linkage programs (52, 54). Finally, partnerships or collaborations among individuals and resources both within secured settings and in the community to support continuity of care should be considered and may address the commonly reported challenge of system navigation or care coordination among individuals with TBI receiving community services (80, 84, 92, 93).

### 4.2. Recommendations for future research

#### 4.2.1. Considerations for sex, gender, and intersecting factors

This scoping review revealed significant and urgent research gaps regarding sex, gender, and intersecting factors. First, information on the race or ethnicity of the participants was documented in only ten articles (51–54, 58, 68–72), with the most common race or ethnicity described as “white” (52–54, 58, 68–72). None of these articles, or any other article included in this review, described if or how rehabilitation interventions or programs explicitly considered racism or the experiences of diverse populations in relation to race, ethnicity, or culture. Given racial/ethnic disparities in the CJS (94), this is a significant gap in knowledge of how and if the rehabilitation interventions described in this study are appropriate for individuals of different race, ethnicity, or culture. Secondly, none of the studies integrated sex and gender-based analyses. Apart from case studies, six articles included only males or men in their study (51–53, 56, 63, 65) and overall, the majority of participants were males or men (ranging from 56 to 100%). None of these studies discussed their findings in relation to sex or gender, or if and how these findings may be applicable to both males and females or gender diverse individuals, with only one article reporting the inclusion of individuals of “other sex” (68). Furthermore, despite the high prevalence of TBI reported in a systematic review specifically on female incarcerated individuals (up to 49% among youths and up to 95% among adults) (10), only one article focused exclusively on female prisoners (54). This article highlighted the unique experiences and needs of women and acknowledged the importance of trauma-informed practice training for prison staff, individual trauma-focused work, and consideration of the service provider's gender, as majority of the participants sustained their TBI through intimate partner violence (54). Research that includes participants across sex and gender are urgently needed to inform gender-transformative care for individuals with TBI who intersect with the CJS.

Overall, the consideration of sex, gender, and intersecting factors in research and rehabilitation is critical because they all contribute to unique experiences that cannot be addressed by looking at a single facet of identity. For example, these experiences and inequities may impact the rehabilitation process, including care pathways and community re-integration, of individuals with TBI who intersect with the CJS. As such, future research that considers sex, gender and intersecting factors and is co-created with participants of diverse sex, gender, and intersecting factors is crucial to inform the development of rehabilitation that is appropriate and accounts for the needs and experiences of diverse individuals.

#### 4.2.2. Considerations regarding the CJS context and unexplored parts of the CJS

Future research should also provide considerations for rehabilitation relevant to the CJS context and focus on individuals who intersect with police, court, and parole to identify opportunities to integrate rehabilitation in those parts of the CJS. Even though up to 72.7% of participants in the articles that described general rehabilitation service use had intersected with the CJS, none described considerations for CJS-involvement. Furthermore, among the articles included in this review, only two documented an intersection with police (49, 59), five with court (50, 55, 57, 59, 60), and six with parole (54–57, 65, 68), with most of the rehabilitation interventions being delivered in inpatient or outpatient settings (60, 61, 63, 65, 66, 69, 70, 72) and jail or prison (51–54, 56). Given that all individuals who are represented in the corrections part of the CJS must have proceeded through police and court, and will proceed through parole, opportunities to support individuals with TBI through police, court, and parole settings must be identified. This is important because TBI-related impairments may negatively impact how an individual behaves and concurrently, how their behavior is perceived by CJS staff (14). Focusing on the other parts of the CJS could provide opportunities for education and timely and appropriate rehabilitation for individuals with TBI regardless of where they intersect in the system.

## 5. Strengths and limitations

We acknowledge limitations of our scoping review. First, we acknowledge publication bias despite our attempt to address this by searching for non-English language articles and gray literature reports. Specifically, while we captured non-English language articles from databases, the websites that we searched for gray literature report were all in English language. As such, our search may have missed other relevant non-English gray literature reports. Furthermore, only published gray literature reports were identified and thus, rehabilitation programs or interventions that were never formally reported or presented would not be captured. To mitigate the impact of this specific limitation on our scoping review, we presented our findings to our PAC and sought feedback on rehabilitation services they may be aware of but not captured in this review. Second, we acknowledge that the outcome of the rehabilitation program or intervention was documented in this review only if reported by the article; as such, we are unable to discuss the efficacy of the rehabilitation programs or interventions. Given that positive outcomes such as obtaining and being satisfied with employment (53, 64) and community involvement (64) were documented, the efficacy of rehabilitation interventions for individuals with TBI who intersect with the CJS should be explored in future research. Finally, we acknowledge that the inclusion of a quality appraisal deviates from scoping review methodology (31, 32); however, no articles were eliminated as a result of the quality appraisal and findings were used to inform the discussion of our findings.

Despite the above limitations, there are major strengths of our scoping review. First, this review was guided by scoping review methodology frameworks (31, 32) to address methodological rigor of existing scoping reviews on rehabilitation (95). Second, a protocol was developed and peer-reviewed (30) to facilitate transparent reporting and conduct of this scoping review. The protocol also included the explicit charting of data related to sex, gender, and intersecting factors to inform opportunities for future research and identify existing rehabilitation that considers diverse individuals with TBI who intersect with the CJS. Third, this review considered rehabilitation for individuals with TBI who intersect with all parts of the CJS (i.e., police, court, corrections, and parole). This addressed an existing research and knowledge gap, as most reviews on TBI and the CJS to date focus on identifying the prevalence of TBI, not rehabilitation, and/or are limited to the corrections setting (4–8, 10, 26). Finally, preliminary findings from this scoping review were shared with a PAC consisting of service providers and healthcare professionals in the CJS and brain injury sectors; health administrators, decision-makers, and policy-makers; and researchers and trainees who conduct research on rehabilitation, TBI, and the CJS (47, 48). Their feedback was integrated in the interpretation of findings from this review.

## 6. Conclusion

This scoping review is the first, to the best of our knowledge, to explore rehabilitation programs and/or interventions available to, or used by, individuals with TBI who intersect with all parts of the CJS. More than half of the articles identified in this review described use of rehabilitation interventions without specific information on the rehabilitation intervention. These articles provide evidence that existing rehabilitation interventions, particularly those provided within inpatient and/or outpatient rehabilitation centers, are already serving individuals with TBI with a history of CJS involvement. Opportunities to integrate rehabilitation for individuals with TBI who intersect with the CJS were identified, specifically through TBI screening to facilitate access to appropriate and individualized interventions, including strategies to address TBI impairments; education to increase TBI awareness; and roles and services that link individuals to relevant supports across the continuum of care and CJS involvement. Furthermore, research in collaboration with individuals with lived experience of TBI and CJS involvement to identify and address barriers and facilitators to screening within the CJS context; assess the feasibility of delivering education on TBI in all parts of the CJS and to co-create these educational materials; and identify opportunities to facilitate continuity of care, particularly from CJS settings to the community, are encouraged. Finally, future studies should address the research gaps regarding sex, gender, and intersecting factors to understand how these experiences impact the rehabilitation process. Addressing these research and knowledge gaps will ultimately advance timely and appropriate rehabilitation of TBI for individuals who intersect with the CJS.

## Data availability statement

The original contributions presented in the study are included in the article/Supplementary material, further inquiries can be directed to the corresponding author.

## Author contributions

VC and AC conceptualized this scoping review. VC, MJE, and JB developed the search strategy. VC and MJE formulated the design, completed the analyses for this review, and drafted the manuscript. RS, ZB-D, SS, AL, and ZC screened the articles. SS, AL, and VC charted the data. VC and RS completed quality appraisal of the included articles. All authors critically reviewed the manuscript and approved the final manuscript.
