# Supplementary material for: Rehabilitation among individuals with traumatic brain injury who intersect with the criminal justice system: A scoping review
Source: Front Neurol. 2023 Jan 17;13:1052294. doi: 10.3389/fneur.2022.1052294 (PMC9886883; doi:10.3389/fneur.2022.1052294)
Supplement: Supplementary file 1 [file Data_Sheet_1.pdf]

Search Strategies – ALL

Database: Ovid MEDLINE(R) ALL <1946 to July 02, 2021>
Search Strategy:
----
1 exp PRISONS/ (10724)
2 exp PRISONERS/ (17360)
3 exp CRIMINALS/ (5344)
4 Criminal Law/ or Jurisprudence/ (34822)
5 Judicial Role/ (3463)
6 (jurisprudenc* or ligitat*).tw,kf. (6769)
7 (legal adj (system? or servic*)).tw,kf. (2104)
8 (prisoner* or prison? or imprison*).tw,kf. (19472)
9 (inmate* or convict* or criminal* or offender?).tw,kf. (42331)
10 (correctional adj2 (setting? or service? or units or unit or facility or facilities or institution* or centre* or center*)).tw,kf. (2340)
11 (penal adj2 (setting? or service? or units or unit or facility or facilities or institution* or centre* or center*)).tw,kf. (161)
12 (jail* or penitentiar* or gaol*).tw,kf. (4636)
13 incarcerat*.tw,kf. (12632)
14 (detain* or detention?).tw,kf. (5363)
15 parole?.tw,kf. (750)
16 probation*.tw,kf. (1668)
17 felon*.tw,kf. (792)
18 Police/ (5608)
19 (police or policing).tw,kf. (16269)
20 law enforce*.tw,kf. (5117)
21 forensic*.tw,kf. (49033)
22 forensic psychiatry/ or "commitment of mentally ill"/ or insanity defense/ (15597)
23 (correctional or forensic).jw. (34954)
24 or/1-23 (193267)
25 "Physical and Rehabilitation Medicine"/ (3376)
26 exp rehabilitation/ (321626)
27 rehab*.tw,kf,jw. (243365)
28 telerehab*.tw,kf,jw. (1188)
29 neurorehab*.tw,kf,jw. (7739)
30 rh.fs. (203624)
31 (physiatrist? or physiatry).tw,kf. (1520)
32 Rehabilitation Centers/ (8380)
33 occupational therapy/ (13662)
34 (occupational adj therap*).tw,kf,jw. (15793)
35 physical therapy specialty/ (2921)
36 (physical adj therap*).tw,kf,jw. (27777)
37 physiotherap*.tw,kf,jw. (34855)
38 physio-therapist*.tw,kf,jw. (11)
39 Speech-Language Pathology/ (3267)
40 (speech adj2 (therap* or patholog*)).tw,kf,jw. (10410)
41 Neuropsychology/ (2465)
42 Neuropsycholog*.tw,kf,jw. (78579)
43 Nutritionists/ (1367)
44 (Nutritionist? or Dietician?).tw,kf,jw. (5003)

45 (therap* adj recreation*).tw,kf,jw. (206)
46 child life specialist?.tw,kf. (161)
47 play therapy/ (1166)
48 (play adj therap*).tw,kf. (483)
49 Respite Care/ (1053)
50 respite.tw,kf. (1914)
51 Case Managers/ (205)
52 Case Management/ (10282)
53 case manag*.tw,kf. (14038)
54 exp Social Work/ (17968)
55 social work*.tw,kf,jw. (21294)
56 Forensic Nursing/ (511)
57 (nurse? or nursing).tw,kf,jw. (771161)
58 Community Integration/ (392)
59 (integrat* or reintegrat* or re-integrat* or reentry or re-entry or resettle* or re-settle*).tw,kf. (593190)
60 Aftercare/ (10251)
61 (Aftercare or "after care").tw,kf. (4924)
62 Transitional Care/ (944)
63 "transitional care".tw,kf. (1727)
64 or/25-63 (2021580)
65 exp Brain Injuries/ (72804)
66 exp Brain Injuries, Traumatic/ (17399)
67 exp Brain Concussion/ (9892)
68 Craniocerebral Trauma/ (22598)
69 tbi*2.tw,kf. (29717)
70 mtbi*2.tw,kf. (3296)
71 concuss*.tw,kf. (10854)
72 postconcuss*.tw,kf. (1495)
73 ((head* or brain* or cerebr* or crani* or skull* or intracran*) adj2 (injur* or trauma* or damag* or wound* or swell* or oedema* or edema* or fracture* or contusion* or pressur*)).tw,kf,jw. (185036)
74 ((brain* or cerebr* or intracerebr* or crani* or intracran* or head* or subdural* or epidural* or extradural*) adj (haematoma* or hematoma* or hemorrhag* or haemorrhag* or bleed*)).tw,kf. (56349)
75 exp cognition disorders/ (101231)
76 ((cogniti* or neurocogniti*) adj2 (impair* or dysfunction* or disorder* or declin*)).tw,kf. (124649)
77 or/65-76 (429105)
78 24 and 64 (20125)
79 24 and 77 (4299)
80 24 and 64 and 77 (644)
81 80 not (exp animals/ not humans.sh.) (644)
82 limit 81 to english language (596)
83 81 not 82 (48)

****

Database: Cochrane Central Register of Controlled Trials <2014 to Present>
Search Strategy:
----

1 exp PRISONS/ (132)
2 exp PRISONERS/ (338)
3 exp CRIMINALS/ (124)
4 Criminal Law/ or Jurisprudence/ (128)
5 Judicial Role/ (14)
6 (jurisprudenc* or ligitat*).tw,kw. (61)
7 (legal adj (system? or servic*)).tw,kw. (54)
8 (prisoner* or prison? or imprison*).tw,kw. (1009)
9 (inmate* or convict* or criminal* or offender?).tw,kw. (2161)
10 (correctional adj2 (setting? or service? or units or unit or facility or facilities or institution* or centre* or center*)).tw,kw. (167)
11 (penal adj2 (setting? or service? or units or unit or facility or facilities or institution* or centre* or center*)).tw,kw. (5)
12 (jail* or penitentiar* or gaol*).tw,kw. (347)
13 incarcerat*.tw,kw. (830)
14 (detain* or detention?).tw,kw. (220)
15 parole?.tw,kw. (122)
16 probation*.tw,kw. (229)
17 felon*.tw,kw. (51)
18 Police/ (76)
19 (police or policing).tw,kw. (689)
20 law enforce*.tw,kw. (135)
21 forensic*.tw,kw. (560)
22 forensic psychiatry/ or "commitment of mentally ill"/ or insanity defense/ (116)
23 (correctional or forensic).jw. (155)
24 or/1-23 (4993)
25 "Physical and Rehabilitation Medicine"/ (19)
26 exp rehabilitation/ (25604)
27 rehab*.tw,kw,jw. (46162)
28 telerehab*.tw,kw,jw. (602)
29 neurorehab*.tw,kw,jw. (1322)
30 rh.fs. (18523)
31 (physiatrist? or physiatry).tw,kw. (187)
32 Rehabilitation Centers/ (317)
33 occupational therapy/ (787)
34 (occupational adj therap*).tw,kw,jw. (3273)
35 physical therapy specialty/ (118)
36 (physical adj therap*).tw,kw,jw. (8505)
37 physiotherap*.tw,kw,jw. (17864)
38 physio-therapist*.tw,kw,jw. (8)
39 Speech-Language Pathology/ (71)
40 (speech adj2 (therap* or patholog*)).tw,kw,jw. (1747)
41 Neuropsychology/ (22)
42 Neuropsycholog*.tw,kw,jw. (9577)
43 Nutritionists/ (46)
44 (Nutritionist? or Dietician?).tw,kw,jw. (1526)
45 (therap* adj recreation*).tw,kw,jw. (31)
46 child life specialist?.tw,kw. (39)
47 play therapy/ (69)
48 (play adj therap*).tw,kw. (177)
49 Respite Care/ (15)

50 respite.tw,kw. (167)
51 Case Managers/ (12)
52 Case Management/ (699)
53 case manag*.tw,kw. (2835)
54 exp Social Work/ (224)
55 social work*.tw,kw,jw. (2098)
56 Forensic Nursing/ (4)
57 (nurse? or nursing).tw,kw,jw. (48642)
58 Community Integration/ (15)
59 (integrat* or reintegrat* or re-integrat* or reentry or re-entry or resettle* or re-settle*).tw,kw. (30052)
60 Aftercare/ (640)
61 (Aftercare or "after care").tw,kw. (189166)
62 Transitional Care/ (58)
63 "transitional care".tw,kw. (421)
64 or/25-63 (312374)
65 exp Brain Injuries/ (2241)
66 exp Brain Injuries/ (2241)
67 exp Brain Concussion/ (394)
68 Craniocerebral Trauma/ (332)
69 tbi*2.tw,kw. (3413)
70 mtbi*2.tw,kw. (406)
71 concuss*.tw,kw. (820)
72 postconcuss*.tw,kw. (275)
73 ((head* or brain* or cerebr* or crani* or skull* or intracran*) adj2 (injur* or trauma* or damag* or wound* or swell* or oedema* or edema* or fracture* or contusion* or pressur*)).tw,kw,jw. (14207)
74 ((brain* or cerebr* or intracerebr* or crani* or intracran* or head* or subdural* or epidural* or extradural*) adj (haematoma* or hematoma* or hemorrhag* or haemorrhag* or bleed*)).tw,kw. (8249)
75 exp cognition disorders/ (3820)
76 ((cogniti* or neurocogniti*) adj2 (impair* or dysfunction* or disorder* or declin*)).tw,kw. (18298)
77 or/65-76 (42412)
78 24 and 64 (1563)
79 24 and 77 (105)
80 24 and 64 and 77 (49)
81 "https://clinicaltrials.gov*".so. (203896)
82 "http://www.who.int/trialsearch*".so. (155837)
83 80 not (81 or 82) (29)
84 limit 83 to english language (28)
85 83 not 84 (1)
86 from 85 keep 1 (1)
87 84 or 86 (29)

****

Database: Embase Classic+Embase <1947 to 2021 July 02>
Search Strategy:
----
1 exp PRISON/ (17576)

2 PRISONER/ (18623)
3 Offender/ (16429)
4 Criminal Justine/ or Jurisprudence/ (30000)
5 legal procedure/ or probation/ (1724)
6 (jurisprudenc* or ligitat*).tw,kw. (2844)
7 (legal adj (system? or servic*)).tw,kw. (2789)
8 (prisoner* or prison? or imprison*).tw,kw. (24540)
9 (inmate* or convict* or criminal* or offender?).tw,kw. (56122)
10 (correctional adj2 (setting? or service? or units or unit or facility or facilities or institution* or centre* or center*)).tw,kw. (2839)
11 (penal adj2 (setting? or service? or units or unit or facility or facilities or institution* or centre* or center*)).tw,kw. (286)
12 (jail* or penitentiar* or gaol*).tw,kw. (5802)
13 incarcerat*.tw,kw. (16383)
14 (detain* or detention?).tw,kw. (7521)
15 parole?.tw,kw. (944)
16 probation*.tw,kw. (2288)
17 felon*.tw,kw. (948)
18 exp police/ or detention/ (16698)
19 (police or policing).tw,kw. (21808)
20 law enforce*.tw,kw. (6447)
21 forensic*.tw,kw. (72189)
22 exp forensic medicine/ (61635)
23 (correctional or forensic).jx. (51575)
24 or/1-23 (265449)
25 rehabilitation medicine/ or physical medicine/ (18588)
26 exp rehabilitation/ (445623)
27 rehab*.tw,kw,jx. (379864)
28 telerehab*.tw,kw,jx. (1333)
29 neurorehab*.tw,kw,jx. (11598)
30 rh.fs. (164241)
31 (physiatrist? or physiatry).tw,kw. (2790)
32 Rehabilitation Center/ (17474)
33 occupational therapy/ (26158)
34 (occupational adj therap*).tw,kw,jx. (31626)
35 exp physiotherapy/ (102360)
36 (physical adj therap*).tw,kw,jx. (54756)
37 physiotherap*.tw,kw,jx. (67438)
38 physio-therapist*.tw,kw,jx. (33)
39 "speech and language rehabilitation"/ (770)
40 (speech adj2 (therap* or patholog*)).tw,kw,jx. (16733)
41 Neuropsychology/ (19586)
42 Neuropsycholog*.tw,kw,jx. (110919)
43 Dietitian/ (13795)
44 (Nutritionist? or Dietician?).tw,kw,jx. (9946)
45 (therap* adj recreation*).tw,kw,jx. (365)
46 child life specialist?.tw,kw. (400)
47 play therapy/ (1940)
48 (play adj therap*).tw,kw. (776)
49 Respite Care/ (1193)
50 respite.tw,kw. (2662)

51 Case Manager/ (2075)
52 Case Management/ (12394)
53 case manag*.tw,kw. (18576)
54 Social Work/ (28425)
55 social work*.tw,kw,jx. (31074)
56 Forensic Nursing/ (522)
57 (nurse? or nursing).tw,kw,jx. (864461)
58 Community Integration/ or community reintegration/ (1862)
59 (integrat* or reintegrat* or re-integrat* or reentry or re-entry or resettle* or re-settle*).tw,kw. (729994)
60 Aftercare/ (8552)
61 (Aftercare or "after care").tw,kw. (7386)
62 Transitional Care/ (3735)
63 "transitional care".tw,kw. (2716)
64 or/25-63 (2525147)
65 exp Brain Injury/ (201496)
66 exp traumatic brain injury/ (55501)
67 Brain concussion/ or postconcussion syndrome/ (9372)
68 head injury/ (55394)
69 tbi*2.tw,kw. (49067)
70 mtbi*2.tw,kw. (5361)
71 concuss*.tw,kw. (15496)
72 postconcuss*.tw,kw. (1972)
73 ((head* or brain* or cerebr* or crani* or skull* or intracran*) adj2 (injur* or trauma* or damag* or wound* or swell* or oedema* or edema* or fracture* or contusion* or pressur*)).tw,kw,jx. (262199)
74 ((brain* or cerebr* or intracerebr* or crani* or intracran* or head* or subdural* or epidural* or extradural*) adj (haematoma* or hematoma* or hemorrhag* or haemorrhag* or bleed*)).tw,kw. (86811)
75 exp cognitive defect/ (532897)
76 ((cogniti* or neurocogniti*) adj2 (impair* or dysfunction* or disorder* or declin*)).tw,kw. (192551)
77 or/65-76 (989597)
78 24 and 64 (27461)
79 24 and 77 (8688)
80 24 and 64 and 77 (1301)
81 80 not medline.cr. (1002)
82 81 not (((rat or rats or mouse or mice or swine or porcine or murine or sheep or lambs or pigs or piglets or rabbit or rabbits or cat or cats or dog or dogs or cattle or bovine or monkey or monkeys or trout or marmoset$1).ti. and animal experiment/) or (Animal experiment/ not (human experiment/ or human/))) (996)
83 limit 82 to english language (914)
84 82 not 83 (82)

****

Database: APA PsycInfo <1806 to June Week 4 2021>
Search Strategy:
----
1 exp Correctional Institutions/ (10303)

2 exp Prisoners/ (11681)
3 Criminal Offenders/ (14101)
4 exp Criminal Justice/ or Criminal Law/ (15090)
5 Legal Processes/ or probation/ or Parole/ (17318)
6 (jurisprudenc* or ligitat*).ti,ab. (1223)
7 (legal adj (system? or servic*)).ti,ab. (3833)
8 (prisoner* or prison? or imprison*).ti,ab. (25851)
9 (inmate* or convict* or criminal* or offender?).ti,ab. (81363)
10 (correctional adj2 (setting? or service? or units or unit or facility or facilities or institution* or centre* or center*)).ti,ab. (4042)
11 (penal adj2 (setting? or service? or units or unit or facility or facilities or institution* or centre* or center*)).ti,ab. (411)
12 (jail* or penitentiar* or gaol*).ti,ab. (4789)
13 incarcerat*.ti,ab. (13368)
14 (detain* or detention?).ti,ab. (5879)
15 parole?.ti,ab. (2620)
16 probation*.ti,ab. (4912)
17 felon*.ti,ab. (1598)
18 Police Personnel/ or exp Law Enforcement/ (35812)
19 (police or policing).ti,ab. (24260)
20 law enforce*.ti,ab. (7380)
21 forensic*.ti,ab. (18747)
22 Forensic Psychiatry/ or Forensic Psychology/ (9356)
23 (correctional or forensic).jx. (5820)
24 or/1-23 (164349)
25 exp rehabilitation/ (50782)
26 rehab*.ti,ab,jx. (79721)
27 telerehab*.ti,ab,jx. (173)
28 neurorehab*.ti,ab,jx. (4663)
29 (physiatrist? or physiatry).ti,ab. (137)
30 exp Rehabilitation Centers/ (1133)
31 occupational therapy/ (6535)
32 (occupational adj therap*).ti,ab,jx. (14233)
33 Physical therapy/ (3064)
34 (physical adj therap*).ti,ab,jx. (3750)
35 physiotherap*.ti,ab,jx. (3317)
36 physio-therapist*.ti,ab,jx. (0)
37 Speech Therapists/ (1304)
38 (speech adj2 (therap* or patholog*)).ti,ab,jx. (9707)
39 Neuropsychology/ (20413)
40 Neuropsycholog*.ti,ab,jx. (80231)
41 (Nutritionist? or Dietician?).ti,ab,jx. (756)
42 (therap* adj recreation*).ti,ab,jx. (579)
43 child life specialist?.ti,ab. (115)
44 play therapy/ (3838)
45 (play adj therap*).ti,ab. (3281)
46 Respite Care/ (464)
47 respite.ti,ab. (1689)
48 exp Social Casework/ (19181)
49 Case Management/ (3261)
50 case manag*.ti,ab. (6771)

51 exp Social Workers/ (13106)
52 social work*.ti,ab,jx. (59524)
53 (nurse? or nursing).ti,ab,jx. (125535)
54 Reintegration/ or exp Social Integration/ (7587)
55 (integrat* or reintegrat* or re-integrat* or reentry or re-entry or resettle* or re-settle*).ti,ab. (244274)
56 Aftercare/ (1117)
57 (Aftercare or "after care").ti,ab. (2899)
58 "transitional care".ti,ab. (323)
59 or/25-58 (607708)
60 exp Brain Injuries/ (21388)
61 exp traumatic brain injury/ (20797)
62 Brain concussion/ (2558)
63 head injuries/ (4580)
64 tbi*2.ti,ab. (11730)
65 mtbi*2.ti,ab. (2073)
66 concuss*.ti,ab. (3519)
67 postconcuss*.ti,ab. (842)
68 ((head* or brain* or cerebr* or crani* or skull* or intracran*) adj2 (injur* or trauma* or damag* or wound* or swell* or oedema* or edema* or fracture* or contusion* or pressur*)).ti,ab,jx. (50324)
69 ((brain* or cerebr* or intracerebr* or crani* or intracran* or head* or subdural* or epidural* or extradural*) adj (haematoma* or hematoma* or hemorrhag* or haemorrhag* or bleed*)).ti,ab. (3513)
70 Cognitive Impairment/ or Mild Cognitive Impairment/ (40237)
71 ((cogniti* or neurocogniti*) adj2 (impair* or dysfunction* or disorder* or declin*)).ti,ab. (66962)
72 or/60-71 (130210)
73 24 and 59 and 72 (892)
74 limit 73 to ("column/opinion" or dissertation or editorial) (66)
75 73 not 74 (826)
76 limit 75 to animal (3)
77 limit 75 to human (792)
78 75 not (76 not 77) (826)
79 limit 78 to english language (782)
80 78 not 79 (44)

****

Database: CINAHL Complete (Wednesday July 7th, 2021 12:09 PM)
Search Strategy:

#	Query	Limiters/Expanders	Last Run Via	Results
S1	(MH "Correctional Facilities")	Expanders - Apply equivalent subjects<br>Search modes - Boolean/Phrase	Interface - EBSCOhost Research Databases<br>Search Screen - Advanced Search<br>Database - CINAHL Complete	6,419
S2	(MH "Prisoners")	Expanders - Apply equivalent subjects	Interface - EBSCOhost Research Databases	9,554

		Search modes - Boolean/Phrase	Search Screen - Advanced Search<br>Database - CINAHL Complete	
S3	(MH "Public Offenders+")	Expanders - Apply equivalent subjects<br>Search modes - Boolean/Phrase	Interface - EBSCOhost Research Databases<br>Search Screen - Advanced Search<br>Database - CINAHL Complete	11,090
S4	(MH "Jurisprudence") OR (MH "Criminal Justice")	Expanders - Apply equivalent subjects<br>Search modes - Boolean/Phrase	Interface - EBSCOhost Research Databases<br>Search Screen - Advanced Search<br>Database - CINAHL Complete	8,397
S5	TI ((jurisprudenc* or ligitat*)) OR AB ((jurisprudenc* or ligitat*))	Expanders - Apply equivalent subjects<br>Search modes - Boolean/Phrase	Interface - EBSCOhost Research Databases<br>Search Screen - Advanced Search<br>Database - CINAHL Complete	417
S6	TI ((legal n1 (system* or servic*))) OR AB ((legal n1 (system* or servic*)))	Expanders - Apply equivalent subjects<br>Search modes - Boolean/Phrase	Interface - EBSCOhost Research Databases<br>Search Screen - Advanced Search<br>Database - CINAHL Complete	1,265
S7	TI ((prison* or imprison*)) OR AB ((prison* or imprison*))	Expanders - Apply equivalent subjects<br>Search modes - Boolean/Phrase	Interface - EBSCOhost Research Databases<br>Search Screen - Advanced Search<br>Database - CINAHL Complete	9,342
S8	TI ((inmate* or convict* or criminal* or offender*)) OR AB ((inmate* or convict* or criminal* or offender*))	Expanders - Apply equivalent subjects<br>Search modes - Boolean/Phrase	Interface - EBSCOhost Research Databases<br>Search Screen - Advanced Search<br>Database - CINAHL Complete	19,547
S9	TI ((correctional n2 (setting or settings or service or services or units or unit or facility or facilities or institution* or centre* or center*))) OR AB ((correctional n2 (setting or settings or service or services or units or unit or facility or facilities or institution* or centre* or center*)))	Expanders - Apply equivalent subjects<br>Search modes - Boolean/Phrase	Interface - EBSCOhost Research Databases<br>Search Screen - Advanced Search<br>Database - CINAHL Complete	1,390
S10	TI ((penal n2 (setting or settings or service or	Expanders - Apply equivalent subjects	Interface - EBSCOhost Research Databases	47

	services or units or unit or facility or facilities or institution* or centre* or center*))) OR AB ((penal n2 (setting or settings or service or services or units or unit or facility or facilities or institution* or centre* or center*)))	Search modes - Boolean/Phrase	Search Screen - Advanced Search<br>Database - CINAHL Complete	
S11	TI ((jail* or penitentiar* or gaol*)) OR AB ((jail* or penitentiar* or gaol*))	Expanders - Apply equivalent subjects<br>Search modes - Boolean/Phrase	Interface - EBSCOhost Research Databases<br>Search Screen - Advanced Search<br>Database - CINAHL Complete	2,451
S12	TI incarcerat* OR AB incarcerat*	Expanders - Apply equivalent subjects<br>Search modes - Boolean/Phrase	Interface - EBSCOhost Research Databases<br>Search Screen - Advanced Search<br>Database - CINAHL Complete	5,706
S13	TI (detain* or detention*) OR AB (detain* or detention*)	Expanders - Apply equivalent subjects<br>Search modes - Boolean/Phrase	Interface - EBSCOhost Research Databases<br>Search Screen - Advanced Search<br>Database - CINAHL Complete	2,811
S14	TI parole* OR AB parole*	Expanders - Apply equivalent subjects<br>Search modes - Boolean/Phrase	Interface - EBSCOhost Research Databases<br>Search Screen - Advanced Search<br>Database - CINAHL Complete	579
S15	TI probation* OR AB probation*	Expanders - Apply equivalent subjects<br>Search modes - Boolean/Phrase	Interface - EBSCOhost Research Databases<br>Search Screen - Advanced Search<br>Database - CINAHL Complete	1,025
S16	TI felon* OR AB felon*	Expanders - Apply equivalent subjects<br>Search modes - Boolean/Phrase	Interface - EBSCOhost Research Databases<br>Search Screen - Advanced Search<br>Database - CINAHL Complete	352
S17	TI (police or policing) OR AB (police or policing)	Expanders - Apply equivalent subjects<br>Search modes - Boolean/Phrase	Interface - EBSCOhost Research Databases<br>Search Screen - Advanced Search<br>Database - CINAHL Complete	8,568
S18	TI law enforce* OR AB law enforce*	Expanders - Apply equivalent subjects	Interface - EBSCOhost Research Databases<br>Search Screen - Advanced	2,527

		Search modes - Boolean/Phrase	Search<br>Database - CINAHL Complete	
S19	TI forensic* OR AB forensic*	Expanders - Apply equivalent subjects<br>Search modes - Boolean/Phrase	Interface - EBSCOhost Research Databases<br>Search Screen - Advanced Search<br>Database - CINAHL Complete	10,221
S20	(MH "Police")	Expanders - Apply equivalent subjects<br>Search modes - Boolean/Phrase	Interface - EBSCOhost Research Databases<br>Search Screen - Advanced Search<br>Database - CINAHL Complete	6,419
S21	(MH "Forensic Psychiatry+")	Expanders - Apply equivalent subjects<br>Search modes - Boolean/Phrase	Interface - EBSCOhost Research Databases<br>Search Screen - Advanced Search<br>Database - CINAHL Complete	1,934
S22	SO correctional or forensic	Expanders - Apply equivalent subjects<br>Search modes - Boolean/Phrase	Interface - EBSCOhost Research Databases<br>Search Screen - Advanced Search<br>Database - CINAHL Complete	8,690
S23	S1 OR S2 OR S3 OR S4 OR S5 OR S6 OR S7 OR S8 OR S9 OR S10 OR S11 OR S12 OR S13 OR S14 OR S15 OR S16 OR S17 OR S18 OR S19 OR S20 OR S21 OR S22	Expanders - Apply equivalent subjects<br>Search modes - Boolean/Phrase	Interface - EBSCOhost Research Databases<br>Search Screen - Advanced Search<br>Database - CINAHL Complete	72,193
S24	(MH "Rehabilitation+")	Expanders - Apply equivalent subjects<br>Search modes - Boolean/Phrase	Interface - EBSCOhost Research Databases<br>Search Screen - Advanced Search<br>Database - CINAHL Complete	305,989
S25	(MH "Physical Medicine")	Expanders - Apply equivalent subjects<br>Search modes - Boolean/Phrase	Interface - EBSCOhost Research Databases<br>Search Screen - Advanced Search<br>Database - CINAHL Complete	1,854
S26	TI rehab* OR AB rehab* OR SO rehab*	Expanders - Apply equivalent subjects<br>Search modes - Boolean/Phrase	Interface - EBSCOhost Research Databases<br>Search Screen - Advanced Search<br>Database - CINAHL Complete	166,864

S27	TI telerehab* OR AB telerehab* OR SO telerehab*	Expanders - Apply equivalent subjects Search modes - Boolean/Phrase	Interface - EBSCOhost Research Databases Search Screen - Advanced Search Database - CINAHL Complete	540
S28	TI neurorehab* OR AB neurorehab* OR SO neurorehab*	Expanders - Apply equivalent subjects Search modes - Boolean/Phrase	Interface - EBSCOhost Research Databases Search Screen - Advanced Search Database - CINAHL Complete	5,842
S29	TI ((physiatrist* or physiatry)) OR AB ((physiatrist* or physiatry))	Expanders - Apply equivalent subjects Search modes - Boolean/Phrase	Interface - EBSCOhost Research Databases Search Screen - Advanced Search Database - CINAHL Complete	1,056
S30	(MH "Rehabilitation Centers+")	Expanders - Apply equivalent subjects Search modes - Boolean/Phrase	Interface - EBSCOhost Research Databases Search Screen - Advanced Search Database - CINAHL Complete	8,805
S31	(MH "Occupational Therapy+") or (MH "Occupational Therapists")	Expanders - Apply equivalent subjects Search modes - Boolean/Phrase	Interface - EBSCOhost Research Databases Search Screen - Advanced Search Database - CINAHL Complete	35,572
S32	TI (occupational n1 therap*) OR AB (occupational n1 therap*) OR SO (occupational n1 therap*)	Expanders - Apply equivalent subjects Search modes - Boolean/Phrase	Interface - EBSCOhost Research Databases Search Screen - Advanced Search Database - CINAHL Complete	53,048
S33	(MH "Physical Therapy+") OR (MH "Physical Therapists")	Expanders - Apply equivalent subjects Search modes - Boolean/Phrase	Interface - EBSCOhost Research Databases Search Screen - Advanced Search Database - CINAHL Complete	160,918
S34	TI (physical n1 therap*) OR AB (physical n1 therap*) OR SO (physical n1 therap*)	Expanders - Apply equivalent subjects Search modes - Boolean/Phrase	Interface - EBSCOhost Research Databases Search Screen - Advanced Search Database - CINAHL Complete	48,862
S35	TI physiotherap* OR AB physiotherap* OR SO physiotherap*	Expanders - Apply equivalent subjects Search modes - Boolean/Phrase	Interface - EBSCOhost Research Databases Search Screen - Advanced Search Database - CINAHL Complete	41,034

S36	TI physio-therapist* OR AB physio-therapist* OR SO physio-therapist*	Expanders - Apply equivalent subjects Search modes - Boolean/Phrase	Interface - EBSCOhost Research Databases Search Screen - Advanced Search Database - CINAHL Complete	5
S37	(MH "Speech-Language Pathologists") OR (MH "Speech-Language Pathology Assistants")	Expanders - Apply equivalent subjects Search modes - Boolean/Phrase	Interface - EBSCOhost Research Databases Search Screen - Advanced Search Database - CINAHL Complete	7,897
S38	TI ((speech n2 (therap* or patholog*))) OR AB ((speech n2 (therap* or patholog*))) OR SO ((speech n2 (therap* or patholog*)))	Expanders - Apply equivalent subjects Search modes - Boolean/Phrase	Interface - EBSCOhost Research Databases Search Screen - Advanced Search Database - CINAHL Complete	17,262
S39	(MH "Neuropsychology")	Expanders - Apply equivalent subjects Search modes - Boolean/Phrase	Interface - EBSCOhost Research Databases Search Screen - Advanced Search Database - CINAHL Complete	1,890
S40	TI Neuropsycholog* OR AB Neuropsycholog* OR SO Neuropsycholog	Expanders - Apply equivalent subjects Search modes - Boolean/Phrase	Interface - EBSCOhost Research Databases Search Screen - Advanced Search Database - CINAHL Complete	14,644
S41	(MH "Dietitians")	Expanders - Apply equivalent subjects Search modes - Boolean/Phrase	Interface - EBSCOhost Research Databases Search Screen - Advanced Search Database - CINAHL Complete	5,425
S42	TI ((Nutritionist* or Dietician*)) OR AB ((Nutritionist* or Dietician*)) OR SO ((Nutritionist* or Dietician*))	Expanders - Apply equivalent subjects Search modes - Boolean/Phrase	Interface - EBSCOhost Research Databases Search Screen - Advanced Search Database - CINAHL Complete	2,656
S43	(MH "Recreational Therapy")	Expanders - Apply equivalent subjects Search modes - Boolean/Phrase	Interface - EBSCOhost Research Databases Search Screen - Advanced Search Database - CINAHL Complete	1,804
S44	(MH "Recreational Therapists")	Expanders - Apply equivalent subjects Search modes - Boolean/Phrase	Interface - EBSCOhost Research Databases Search Screen - Advanced Search Database - CINAHL Complete	184

S45	TI (therap* n1 recreation*) OR AB (therap* n1 recreation*) OR SO (therap* n1 recreation*)	Expanders - Apply equivalent subjects<br>Search modes - Boolean/Phrase	Interface - EBSCOhost Research Databases<br>Search Screen - Advanced Search<br>Database - CINAHL Complete	1,642
S46	TI child life specialist* OR AB child life specialist* OR SO child life specialist*	Expanders - Apply equivalent subjects<br>Search modes - Boolean/Phrase	Interface - EBSCOhost Research Databases<br>Search Screen - Advanced Search<br>Database - CINAHL Complete	160
S47	(MH "Play Therapy")	Expanders - Apply equivalent subjects<br>Search modes - Boolean/Phrase	Interface - EBSCOhost Research Databases<br>Search Screen - Advanced Search<br>Database - CINAHL Complete	1,253
S48	TI (play n1 therap*) OR AB (play n1 therap*) OR (play n1 therap*)	Expanders - Apply equivalent subjects<br>Search modes - Boolean/Phrase	Interface - EBSCOhost Research Databases<br>Search Screen - Advanced Search<br>Database - CINAHL Complete	2,283
S49	(MH "Respite Care")	Expanders - Apply equivalent subjects<br>Search modes - Boolean/Phrase	Interface - EBSCOhost Research Databases<br>Search Screen - Advanced Search<br>Database - CINAHL Complete	1,415
S50	TI respite OR AB respite	Expanders - Apply equivalent subjects<br>Search modes - Boolean/Phrase	Interface - EBSCOhost Research Databases<br>Search Screen - Advanced Search<br>Database - CINAHL Complete	1,769
S51	(MH "Case Managers")	Expanders - Apply equivalent subjects<br>Search modes - Boolean/Phrase	Interface - EBSCOhost Research Databases<br>Search Screen - Advanced Search<br>Database - CINAHL Complete	4,177
S52	(MH "Case Management")	Expanders - Apply equivalent subjects<br>Search modes - Boolean/Phrase	Interface - EBSCOhost Research Databases<br>Search Screen - Advanced Search<br>Database - CINAHL Complete	17,882
S53	TI "case manag*" OR AB "case manag*"	Expanders - Apply equivalent subjects<br>Search modes - Boolean/Phrase	Interface - EBSCOhost Research Databases<br>Search Screen - Advanced Search<br>Database - CINAHL Complete	11,743

S54	(MH "Social Work+")	Expanders - Apply equivalent subjects<br>Search modes - Boolean/Phrase	Interface - EBSCOhost Research Databases<br>Search Screen - Advanced Search<br>Database - CINAHL Complete	13,910
S55	(MH "Social Workers")	Expanders - Apply equivalent subjects<br>Search modes - Boolean/Phrase	Interface - EBSCOhost Research Databases<br>Search Screen - Advanced Search<br>Database - CINAHL Complete	9,624
S56	TI "social work*" OR AB "social work*" OR SO "social work*"	Expanders - Apply equivalent subjects<br>Search modes - Boolean/Phrase	Interface - EBSCOhost Research Databases<br>Search Screen - Advanced Search<br>Database - CINAHL Complete	40,333
S57	(MH "Forensic Nursing")	Expanders - Apply equivalent subjects<br>Search modes - Boolean/Phrase	Interface - EBSCOhost Research Databases<br>Search Screen - Advanced Search<br>Database - CINAHL Complete	1,778
S58	(MH "Correctional Health Nursing")	Expanders - Apply equivalent subjects<br>Search modes - Boolean/Phrase	Interface - EBSCOhost Research Databases<br>Search Screen - Advanced Search<br>Database - CINAHL Complete	1,208
S59	TI ((nurse or nurses or nursing)) OR AB ((nurse or nurses or nursing)) OR SO ((nurse or nurses or nursing))	Expanders - Apply equivalent subjects<br>Search modes - Boolean/Phrase	Interface - EBSCOhost Research Databases<br>Search Screen - Advanced Search<br>Database - CINAHL Complete	1,214,252
S60	(MH "Community Reintegration")	Expanders - Apply equivalent subjects<br>Search modes - Boolean/Phrase	Interface - EBSCOhost Research Databases<br>Search Screen - Advanced Search<br>Database - CINAHL Complete	2,039
S61	TI ((integrat* or reintegrat* or re-integrat* or reentry or re-entry or resettle* or re-settle)) OR AB ((integrat* or reintegrat* or re-integrat* or reentry or re-entry or resettle* or re-settle))	Expanders - Apply equivalent subjects<br>Search modes - Boolean/Phrase	Interface - EBSCOhost Research Databases<br>Search Screen - Advanced Search<br>Database - CINAHL Complete	139,825
S62	(MH "After Care")	Expanders - Apply equivalent subjects<br>Search modes - Boolean/Phrase	Interface - EBSCOhost Research Databases<br>Search Screen - Advanced Search	16,201

			Database - CINAHL Complete	
S63	TI ((Aftercare or "after care")) OR AB ((Aftercare or "after care"))	Expanders - Apply equivalent subjects<br>Search modes - Boolean/Phrase	Interface - EBSCOhost Research Databases<br>Search Screen - Advanced Search<br>Database - CINAHL Complete	1,643
S64	TI "transitional care" OR AB "transitional care"	Expanders - Apply equivalent subjects<br>Search modes - Boolean/Phrase	Interface - EBSCOhost Research Databases<br>Search Screen - Advanced Search<br>Database - CINAHL Complete	1,352
S65	(MH "Transitional Care")	Expanders - Apply equivalent subjects<br>Search modes - Boolean/Phrase	Interface - EBSCOhost Research Databases<br>Search Screen - Advanced Search<br>Database - CINAHL Complete	2,153
S66	S24 OR S25 OR S26 OR S27 OR S28 OR S29 OR S30 OR S31 OR S32 OR S33 OR S34 OR S35 OR S36 OR S37 OR S38 OR S39 OR S40 OR S41 OR S42 OR S43 OR S44 OR S45 OR S46 OR S47 OR S48 OR S49 OR S50 OR S51 OR S52 OR S53 OR S54 OR S55 OR S56 OR S57 OR S58 OR S59 OR S60 OR S61 OR S62 OR S63 OR S64 OR S65	Expanders - Apply equivalent subjects<br>Search modes - Boolean/Phrase	Interface - EBSCOhost Research Databases<br>Search Screen - Advanced Search<br>Database - CINAHL Complete	1,861,849
S67	(MH "Brain Injuries+")	Expanders - Apply equivalent subjects<br>Search modes - Boolean/Phrase	Interface - EBSCOhost Research Databases<br>Search Screen - Advanced Search<br>Database - CINAHL Complete	30,678
S68	(MH "Head Injuries")	Expanders - Apply equivalent subjects<br>Search modes - Boolean/Phrase	Interface - EBSCOhost Research Databases<br>Search Screen - Advanced Search<br>Database - CINAHL Complete	7,736
S69	(MH "Brain Concussion+")	Expanders - Apply equivalent subjects<br>Search modes - Boolean/Phrase	Interface - EBSCOhost Research Databases<br>Search Screen - Advanced Search<br>Database - CINAHL Complete	5,665

S70	TI (TBI* OR mTBI*) OR AB (TBI* OR mTBI*)	Expanders - Apply equivalent subjects<br>Search modes - Boolean/Phrase	Interface - EBSCOhost Research Databases<br>Search Screen - Advanced Search<br>Database - CINAHL Complete	10,203
S71	TI (concuss* or postconcuss*) OR AB (concuss* or postconcuss*)	Expanders - Apply equivalent subjects<br>Search modes - Boolean/Phrase	Interface - EBSCOhost Research Databases<br>Search Screen - Advanced Search<br>Database - CINAHL Complete	5,904
S72	TI (((head* or brain* or cerebr* or crani* or skull* or intracran*) n2 (injur* or trauma* or damag* or wound* or swell* or oedema* or edema* or fracture* or contusion* or pressur*))) OR AB (((head* or brain* or cerebr* or crani* or skull* or intracran*) n2 (injur* or trauma* or damag* or wound* or swell* or oedema* or edema* or fracture* or contusion* or pressur*))) OR SO (((head* or brain* or cerebr* or crani* or skull* or intracran*) n2 (injur* or trauma* or damag* or wound* or swell* or oedema* or edema* or fracture* or contusion* or pressur*)))	Expanders - Apply equivalent subjects<br>Search modes - Boolean/Phrase	Interface - EBSCOhost Research Databases<br>Search Screen - Advanced Search<br>Database - CINAHL Complete	51,213
S73	TI (((brain* or cerebr* or intracerebr* or crani* or intracran* or head* or subdural* or epidural* or extradural*) n1 (haematoma* or hematoma* or hemorrhag* or haemorrhag* or bleed*))) OR AB (((brain* or cerebr* or intracerebr* or crani* or intracran* or head* or subdural* or epidural* or extradural*) n1 (haematoma* or	Expanders - Apply equivalent subjects<br>Search modes - Boolean/Phrase	Interface - EBSCOhost Research Databases<br>Search Screen - Advanced Search<br>Database - CINAHL Complete	13,257

	hematoma* or hemorrhag* or haemorrhag* or bleed*)))			
S74	(MH "Cognition Disorders+")	Expanders - Apply equivalent subjects<br>Search modes - Boolean/Phrase	Interface - EBSCOhost Research Databases<br>Search Screen - Advanced Search<br>Database - CINAHL Complete	33,382
S75	TI (((cogniti* or neurocogniti*) n2 (impair* or dysfunction* or disorder* or declin*))) OR AB (((cogniti* or neurocogniti*) n2 (impair* or dysfunction* or disorder* or declin*)))	Expanders - Apply equivalent subjects<br>Search modes - Boolean/Phrase	Interface - EBSCOhost Research Databases<br>Search Screen - Advanced Search<br>Database - CINAHL Complete	43,852
S76	S67 OR S68 OR S69 OR S70 OR S71 OR S72 OR S73 OR S74 OR S75	Expanders - Apply equivalent subjects<br>Search modes - Boolean/Phrase	Interface - EBSCOhost Research Databases<br>Search Screen - Advanced Search<br>Database - CINAHL Complete	132,397
S77	S23 AND S66 AND S76	Expanders - Apply equivalent subjects<br>Search modes - Boolean/Phrase	Interface - EBSCOhost Research Databases<br>Search Screen - Advanced Search<br>Database - CINAHL Complete	364
S78	S77	Limiters - English Language<br>Expanders - Apply equivalent subjects<br>Search modes - Boolean/Phrase	Interface - EBSCOhost Research Databases<br>Search Screen - Advanced Search<br>Database - CINAHL Complete	359
S79	S77 NOT S78	Expanders - Apply equivalent subjects<br>Search modes - Boolean/Phrase	Interface - EBSCOhost Research Databases<br>Search Screen - Advanced Search<br>Database - CINAHL Complete	5

Database: Criminal Justice Abstracts (Wednesday, July 7, 2021)
Search Strategy:

#	Query	Limiters/Expanders	Last Run Via	Results
S1	(TI rehab* OR AB rehab* OR SO rehab*) OR (TI neurorehab* OR AB neurorehab* OR SO neurorehab*) OR (TI telerehab* OR AB	Expanders - Apply equivalent subjects<br>Search modes - Boolean/Phrase	Interface - EBSCOhost Research Databases<br>Search Screen - Advanced Search<br>Database - Criminal Justice Abstracts with Full Text	9,678

	telerehab* OR SO telerehab*) OR (TI ((physiatrist* or physiatry)) OR AB ((physiatrist* or physiatry)))			
S2	(TI (occupational n1 therap*) OR AB (occupational n1 therap*) OR SO (occupational n1 therap*)) OR (TI (physical n1 therap*) OR AB (physical n1 therap*) OR SO (physical n1 therap*)) OR (TI physiotherap* OR AB physiotherap* OR SO physiotherap* TI physio-therapist* OR AB physio-therapist* OR SO physio-therapist*) OR (TI ((speech n2 (therap* or patholog*))) OR AB ((speech n2 (therap* or patholog*))) OR SO ((speech n2 (therap* or patholog*)))) OR (TI Neuropsycholog* OR AB Neuropsycholog* OR SO Neuropsycholog) OR (TI ((Nutritionist* or Dietician*)) OR AB ((Nutritionist* or Dietician*)) OR SO ((Nutritionist* or Dietician*))) OR (TI (therap* n1 recreation*) OR AB (therap* n1 recreation*) OR SO (therap* n1 recreation*)) OR (TI child life specialist* OR AB child life specialist* OR SO child life specialist*) OR (TI (play n1 therap*) OR AB (play n1 therap*) OR (play n1 therap*)) OR (TI respite OR AB respite) OR (TI "case manag*" OR AB "case manag*") OR (TI "social work*" OR AB "social work*" OR SO "social work*")	Expanders - Apply equivalent subjects<br>Search modes - Boolean/Phrase	Interface - EBSCOhost Research Databases<br>Search Screen - Advanced Search<br>Database - Criminal Justice Abstracts with Full Text	21,450
S3	(TI ((nurse or nurses or nursing)) OR AB ((nurse or nurses or nursing)) OR SO ((nurse or nurses or nursing))) OR (TI ((integrat* or reintegrat* or re-integrat* or reentry or re-	Expanders - Apply equivalent subjects<br>Search modes - Boolean/Phrase	Interface - EBSCOhost Research Databases<br>Search Screen - Advanced Search<br>Database - Criminal Justice Abstracts with Full Text	24,812

	entry or resettle* or re-settle)) OR AB ((integrat* or reintegrat* or re-integrat* or reentry or re-entry or resettle* or re-settle))) OR (TI ((Aftercare or "after care")) OR AB ((Aftercare or "after care"))) OR (TI "transitional care" OR AB "transitional care")			
S4	S1 OR S2 OR S3	Expanders - Apply equivalent subjects<br>Search modes - Boolean/Phrase	Interface - EBSCOhost Research Databases<br>Search Screen - Advanced Search<br>Database - Criminal Justice Abstracts with Full Text	52,837
S5	(TI (TBI* OR mTBI*) OR AB (TBI* OR mTBI*)) OR (TI (concuss* or postconcuss*) OR AB (concuss* or postconcuss*)) OR (TI (((head* or brain* or cerebr* or crani* or skull* or intracran*) n2 (injur* or trauma* or damag* or wound* or swell* or oedema* or edema* or fracture* or contusion* or pressur*))) OR AB (((head* or brain* or cerebr* or crani* or skull* or intracran*) n2 (injur* or trauma* or damag* or wound* or swell* or oedema* or edema* or fracture* or contusion* or pressur*))) OR SO (((head* or brain* or cerebr* or crani* or skull* or intracran*) n2 (injur* or trauma* or damag* or wound* or swell* or oedema* or edema* or fracture* or contusion* or pressur*)))) OR (TI (((brain* or cerebr* or intracerebr* or crani* or intracran* or head* or subdural* or epidural* or extradural*) n1 (haematoma* or hematoma* or hemorrhag* or haemorrhag* or bleed*))) OR AB (((brain* or cerebr* or intracerebr* or crani* or intracran* or	Expanders - Apply equivalent subjects<br>Search modes - Boolean/Phrase	Interface - EBSCOhost Research Databases<br>Search Screen - Advanced Search<br>Database - Criminal Justice Abstracts with Full Text	3,118

	head* or subdural* or epidural* or extradural*) n1 (haematoma* or hematoma* or hemorrhag* or haemorrhag* or bleed*)))) OR (TI (((cogniti* or neurocogniti*) n2 (impair* or dysfunction* or disorder* or declin*))) OR AB (((cogniti* or neurocogniti*) n2 (impair* or dysfunction* or disorder* or declin*))))			
S6	S4 AND S5	Expanders - Apply equivalent subjects<br>Search modes - Boolean/Phrase	Interface - EBSCOhost Research Databases<br>Search Screen - Advanced Search<br>Database - Criminal Justice Abstracts with Full Text	367
S7	S6	Expanders - Apply equivalent subjects<br>Search modes - Boolean/Phrase	Interface - EBSCOhost Research Databases<br>Search Screen - Advanced Search<br>Database - Criminal Justice Abstracts with Full Text	367
S8	S6	Expanders - Apply equivalent subjects<br>Narrow by Language: - english<br>Search modes - Boolean/Phrase	Interface - EBSCOhost Research Databases<br>Search Screen - Advanced Search<br>Database - Criminal Justice Abstracts with Full Text	363
S9	S7 NOT S8	Expanders - Apply equivalent subjects<br>Search modes - Boolean/Phrase	Interface - EBSCOhost Research Databases<br>Search Screen - Advanced Search<br>Database - Criminal Justice Abstracts with Full Text	4

Database: Nursing and Allied Health Premium (Wednesday July 7, 2021)
Search Strategy:

208 results (208 English, 0 other)
(NOFT(jurisprudenc* OR ligitat*) OR NOFT(legal n/1 system*) OR NOFT(legal n/1 servic*) OR NOFT(prison* OR imprison* OR inmate* OR convict* OR criminal* OR offender* OR jail* OR penitentiar* OR gaol*) OR NOFT(correctional n/2 (setting or settings or service or services or units or unit or facility or facilities or institution* or centre* or center*)) OR NOFT(penal n2 (setting OR settings OR service OR services OR units OR unit OR facility OR facilities OR institution* OR centre* OR center*)) OR NOFT(incarcerat* OR detain* OR detention* OR parole* OR probation* OR police OR policing OR forensic*) OR NOFT("law enforce*") OR JN(correctional OR forensic)) AND (NOFT(TBI* OR mTBI* OR concuss* OR postconcuss*) OR NOFT((head* or brain* or cerebr* or crani* or skull* or intracran*) n/2 (injur* or trauma* or damag* or wound* or swell* or oedema* or edema* or fracture* or contusion* or pressur*)) OR PUB((head* or brain* or cerebr* or crani* or skull* or intracran*) n/2 (injur* or trauma* or damag* or wound* or swell* or oedema* or edema* or fracture* or contusion* or pressur*)) OR

NOFT((brain* or cerebr* or intracerebr* or crani* or intracran* or head* or subdural* or epidural* or extradural*) n/1 (haematoma* or hematoma* or hemorrhag* or haemorrhag* or bleed*)) OR NOFT((cogniti* or neurocogniti*) n/2 (impair* or dysfunction* or disorder* or declin*))) AND (NOFT(rehab* OR telerehab* OR neurorehab* OR physiatrist* OR physiatry OR Neuropsycholog* OR Nutritionist* or Dietician* OR respite) OR PUB(rehab* OR telerehab* OR neurorehab* OR Neuropsycholog* OR Nutritionist* or Dietician* OR Respite) OR NOFT(occupational n/1 therap*) OR PUB(occupational n/1 therap*) OR NOFT(physical n1 therap*) OR PUB(physical n/1 therap*) OR NOFT(physiotherap* or "physio-therap*") OR PUB(physiotherap* or "physio-therap*") OR NOFT(speech n/2 (therap* or patholog*)) OR PUB(speech n/2 (therap* or patholog*)) OR NOFT(therap* n/1 recreation*) OR PUB(therap* n/1 recreation*) OR NOFT("child life specialist*") OR NOFT(play n/1 therap*) OR NOFT("case manag*") OR NOFT("social work*") OR NOFT(nurse or nurses or nursing) Or NOFT(integrat* or reintegrat* or "re-integrat*" or reentry or "re-entry" or resettle* or "re-settle") OR NOFT(Aftercare or "after care") OR NOFT("transitional care"))

Database: Applied Social Sciences Index & Abstracts (ASSIA) (Wednesday July 7, 2021)
Search Strategy:

130 results (130 English; 0 Other):
(NOFT(jurisprudenc* OR ligitat*) OR NOFT(legal n/1 system*) OR NOFT(legal n/1 servic*) OR NOFT(prison* OR imprison* OR inmate* OR convict* OR criminal* OR offender* OR jail* OR penitentiar* OR gaol*) OR NOFT(correctional n/2 (setting or settings or service or services or units or unit or facility or facilities or institution* or centre* or center*)) OR NOFT(penal n2 (setting OR settings OR service OR services OR units OR unit OR facility OR facilities OR institution* OR centre* OR center*)) OR NOFT(incarcerat* OR detain* OR detention* OR parole* OR probation* OR police OR policing OR forensic*) OR NOFT("law enforce*") OR JN(correctional OR forensic)) AND (NOFT(TBI* OR mTBI* OR concuss* OR postconcuss*) OR NOFT((head* or brain* or cerebr* or crani* or skull* or intracran*) n/2 (injur* or trauma* or damag* or wound* or swell* or oedema* or edema* or fracture* or contusion* or pressur*)) OR PUB((head* or brain* or cerebr* or crani* or skull* or intracran*) n/2 (injur* or trauma* or damag* or wound* or swell* or oedema* or edema* or fracture* or contusion* or pressur*)) OR NOFT((brain* or cerebr* or intracerebr* or crani* or intracran* or head* or subdural* or epidural* or extradural*) n/1 (haematoma* or hematoma* or hemorrhag* or haemorrhag* or bleed*)) OR NOFT((cogniti* or neurocogniti*) n/2 (impair* or dysfunction* or disorder* or declin*))) AND (NOFT(rehab* OR telerehab* OR neurorehab* OR physiatrist* OR physiatry OR Neuropsycholog* OR Nutritionist* or Dietician* OR respite) OR PUB(rehab* OR telerehab* OR neurorehab* OR Neuropsycholog* OR Nutritionist* or Dietician* OR Respite) OR NOFT(occupational n/1 therap*) OR PUB(occupational n/1 therap*) OR NOFT(physical n1 therap*) OR PUB(physical n/1 therap*) OR NOFT(physiotherap* or "physio-therap*") OR PUB(physiotherap* or "physio-therap*") OR NOFT(speech n/2 (therap* or patholog*)) OR PUB(speech n/2 (therap* or patholog*)) OR NOFT(therap* n/1 recreation*) OR PUB(therap* n/1 recreation*) OR NOFT("child life specialist*") OR NOFT(play n/1 therap*) OR NOFT("case manag*") OR NOFT("social work*") OR NOFT(nurse or nurses or nursing) Or NOFT(integrat* or reintegrat* or "re-integrat*" or reentry or "re-entry" or resettle* or "re-settle") OR NOFT(Aftercare or "after care") OR NOFT("transitional care"))

Grey Literature
Reports from the following brain injury, criminal justice system, and rehabilitation organizations were searched:

- ABI Justice
- Acquired Brain Injury Ireland
- American Academy of Physical Medicine and Rehabilitation
- Australian Capital Territory Corrective Services
- Barrow Cadbury Trust
- Brain and Spine Foundation
- Brain Injury Association of America
- Brain Injury Australia (BIA)
- Brain Injury Canada+ Provincial and local brain injury associations identified therein
- Brain Injury New Zealand
- Brain Injury Rehabilitation Trust (BIRT)
- Brain Research UK
- Brain Trauma Foundation
- British Society of Rehabilitation Medicine
- Canadian Association of Elizabeth Fry
- Canadian Association of Physical Medicine and Rehab
- Canadian Mental Health Association
- Centre for Crime and Justice Studies
- Cheshire and Merseyside Rehabilitation Network
- Child Brain Injury Trust
- Concussions Ontario
- Connectivity Traumatic Brain Injury Australia
- Correctional Service Canada
- Criminal Justice Alliance
- Department of Corrections (New Zealand)
- Disability Federation of Ireland
- Disability Services USAGov
- Enable Ireland
- European Brain Injury Society
- Federal Bureau of Prisons
- GTA Rehab Network
- Headway
- Headway : Brain Injury Auckland New Zealand
- Headway Ireland
- Her Majesty's Prison and Probation Service
- Human Services and Justice Coordinating Committee
- International Brain Injury Association

- Irish Penal Reform Trust
- Irish Prison Service
- John Howard Society
- John Howard Society of Ontario
- Justice (UK charity)
- JustSpeak
- Kessler Foundation
- Law and Justice Foundation of NSW
- MacArthur Foundation
- March of Dimes Canada
- Mental Health Commission of Canada
- Mental Health Foundation (UK)
- Mental Health Ireland
- Ministry of Health Disability Services
- National Institute of Mental Health (NIMH)
- National Mental Health Consumer and Carer Forum
- New South Wales Corrective Services
- New Zealand Parole Board
- Northern Territory Correctional Services
- Office for Disability Issues
- Office of Disability Employment Policy (ODEP)
- Parole Board (Ireland)
- Parole Board of Canada
- People with Disabilities Act
- Probation Officers Association of Ontario
- PSR RPS Canada
- Queensland Corrective Services
- Rehabilitation Services Administration (RSA)
- SameYou
- South Australia Department for Correctional Services
- Synapse
- Tasmania Corrective Services
- The Center on Brain Injury Research & Training
- The Disabilities Trust
- The Parole Board (UK)
- The Sentencing Project
- Toronto ABI Network
- Toronto Rehabilitation Institute
- Traumatic Brain Injury Center of Excellence
- U.S. Department of Justice
- United Kingdom Acquired Brain Injury Forum

- Victoria Corrections Prisons and Parole
- Western Australia Department of Corrective Services
- Women with Disabilities Victoria
