# Supplementary material for: Rehabilitation among individuals with traumatic brain injury who intersect with the criminal justice system: A scoping review
Source: Front Neurol. 2023 Jan 17;13:1052294. doi: 10.3389/fneur.2022.1052294 (PMC9886883; doi:10.3389/fneur.2022.1052294)
Supplement: Supplementary file 2 [file Data_Sheet_2.pdf]

Study (Author, Year, Country)	Study Design & Objective	Study Sample[a]	TBI Status	Criminal Justice Status	Rehabilitation Intervention, Team, Outcome	TBI-Specific Facilitators, Barriers, Gaps
Bannon et al., 2020<br><br>United States	Cohort Study<br><br>To compare characteristics of those who do and do not sustain subsequent TBIs following index TBI and to identify reinjury risk factors	N= 11,353<br>- Age [Years]: 16-20 (15.4%), 21-29 (23.6%), 30-39 (16.1%), 40-49 (15.4%), 50-59 (12.6%), 60+ (17.0%)<br>- Sex [Males]: 73.0%<br>- Race: White (67.6%), Black (17.4%), Hispanic (10.5%), Other (4.5%)<br>- Employment: Unemployed (11.8%), employed (88.2%)<br>- Education: Some HS or less (23.5%), HS/GED/trade (35.3%), some college/associates (24.2%), college degree (10.9%), advanced degree (5.4%)<br>- Marital status: Single (47.9%), Married (32.4%), Divorced/separated/widowed (19.7%), Missing (0.2%)	- Screening: OSU-TBI-ID<br><br>- One index TBI (91.8%)<br>- ≥1 additional TBI w/LOC or altered consciousness (7.9%)<br>- Injury Severity (GCS Score): mild (38.3%), moderate (15.9%), severe (46.7%), missing (24.0%)	- Intersection: Corrections<br><br>- Incarceration hx (9.0%)	Intervention:<br>- NR<br>- Location: Inpatient rehabilitation<br><br>Rehabilitation Team:<br>- NR<br><br>Outcome:<br>- N/A to rehabilitation intervention<br><br>Funding of Rehabilitation Program/Intervention:<br>- NR	Barriers:<br>- NR<br><br>Facilitators:<br>- NR<br><br>Gaps:<br>- NR
Bezeau et al., 2004<br><br>Canada	Case Study<br><br>Discuss types of brain damage that commonly lead to sexually intrusive behaviour, provide guidance for assessment, and present a 3-stage tx model	- 31 yr old man (referred to as "he/his")<br>- Student in final yr of university when he sustained his TBI<br>- Employed prior to TBI<br>- Long-term relationship prior to TBI	- GCS: 3<br>- Fell into a shallow gully, struck his head<br>- Diagnosed w/severe TBI w/diffuse axonal injury<br>- Remained in a coma for 5 wks and left the hospital in 4 mos	- Intersection: Policing, corrections<br><br>- Arrested for sexual misconduct (sexually harassing and attempting to touch two teenage girls)	Intervention:<br>- Elementary Level: behavioural, environmental, and pharmacological interventions<br>- Intermediate Level: psychoeducation and selective cognitive-behavioural intervention<br>- Advanced Level: modified relapse prevention or advanced cognitive-behavioural program<br><br>Rehabilitation Team:<br>- Psychosocial counsellor, neuropsychiatric consultant, psychoeducation specialist, community placement personnel, social workers, "community caregivers", primary resource workers, neuroradiologist<br><br>Outcome:	Barriers:<br>- NR<br><br>Facilitators:<br>- Comprehensive assessment and treatment, including involvement of different professions, pharmacological intervention, role modelling by primary resource workers, active participation in transition process<br><br>Gaps:<br>- NR

Study (Author, Year, Country)	Study Design & Objective	Study Sample[a]	TBI Status	Criminal Justice Status	Rehabilitation Intervention, Team, Outcome	TBI-Specific Facilitators, Barriers, Gaps
					- 6 mos follow-up after transition to community – continues to function well in gp home environment in home community w/no reported incidences of sexually intrusive behaviour<br><br>Funding of Rehabilitation Program/Intervention:<br>- NR	
Chitsabesan et al., 2015<br><br>United Kingdom	Cohort Study<br><br>To describe (1) the profile of juvenile offenders w/TBI and associated comorbidity w/other neurodevelopmental disorders, mental health needs, and offending behavior and (2) the development of a specialist brain injury service for juvenile offenders w/TBI w/in custody	N= 93<br>- Age [Mean ± SD]: 16.9 ± 0.6<br>- Sex [Males]: 100.0%<br>- Race/ethnicity: White British (90.0%), African Caribbean (2.0%), mixed race (8.0%)	- Screening: CHAT<br>- 82.0% "sustained brain injury that resulted in them being knocked out or dazed and confused"<br>- Mean ± SD # of TBI/person: 2.4 ± 2.0<br>- 61% sought medical attention<br>- 22% remained in hospital following assessment	- Intersection: Corrections<br><br>- Serving a custodial sentence (86.0%)<br>- First time in custody (41.0%)<br><br>Offences:<br>- Theft (burglary or robberies): (47.0%)<br>- Drug-related/public order offences (7.0%)<br><br>Violent offences (62.0%):<br>- Aggravated robbery (31.0%)<br>- Assault (26.0%)<br>Sexual offences (3.0%)<br>- Attempted murder (2.0%)	Intervention:<br>- Brain Injury Linkworker Service providing specialized support for young people with TBI<br>- Screening for health needs, TBI, impairments, developmental hx, and comorbid needs<br>- Assessment (neurocognitive tests) to develop personalized goals, develop summary report, and identify recommendations<br>- Interventions to support individual, including education on TBI, behavioural strategies, coping strategies and techniques, specialist advice regarding impact of TBI from Linkworker to other professionals working with the individual<br>- D/c planning & community interventions: interventions adapted in preparation for release, care plan and discharge summary developed, referral to specialist brain injury service, ongoing supports from Linkworker 8 weeks post-release (e.g., helping them organize and attend appointments, reengage with education and training and community services for additional support)<br><br>Rehabilitation Team:<br>- Linkworker, education staff, mental health nurse, key worker<br><br>Outcome:	Barriers:<br>- NR<br><br>Facilitators:<br>- NR<br><br>Gaps:<br>- NR

Study (Author, Year, Country)	Study Design & Objective	Study Sample[a]	TBI Status	Criminal Justice Status	Rehabilitation Intervention, Team, Outcome	TBI-Specific Facilitators, Barriers, Gaps
					- N/A to rehabilitation intervention<br><br>Funding of Rehabilitation Program/Intervention:<br>- "Primarily funded by the Disabilities Trust Foundation"	
Dillahunt-Aspillaga et al., 2015<br><br>United Kingdom	Cohort Study<br><br>To examine predictors of behavioural service use, incarceration, and associated expenditures for individuals w/TBI	N= 910<br>- Age [Years]: >26 (19.0%), 26-64 (81.0%)<br>- Gender [Males]: 58.0%<br>- Race: Black (12.3%), Hispanic (3.2%), White (53.8%), Other (30.7%)	- 100.0% TBI (ICD-9 codes)	- Intersection: Corrections<br><br>- 25.1% CJS encounters, identified in the Florida Dept Law Enforcement Arrest dataset, Pinellas County Criminal Justice Information System, Florida Dept of Corrections (prison) data, and Florida Dept of Juvenile Justice System	Intervention:<br>- NR (participants were identified in databases for mental health and substance abuse)<br>- Study assessed behavioural health service use<br><br>Rehabilitation Team:<br>- NR<br><br>Outcome:<br>- 23.1% mental health service use<br>- 8.5% substance abuse service use<br>- 6.2% mental health and substance abuse service use<br>- CJS involvement associated w/high public expenditure (HR=7.187, CI=4.621-11.176)<br>- Receiving mental health services associated w/high public expenditure (HR=25.245)<br>- Receiving substance abuse services associated w/low public expenditure (HR=0.589)<br><br>Funding of Rehabilitation Program/Intervention:<br>- NR	Barriers:<br>- NR<br><br>Facilitators:<br>- NR<br><br>Gaps:<br>- NR
Glass et al., 2000<br><br>United States	Cohort Study<br><br>To determine the prevalence and types of cognitive impairment, and detect	N= 134<br>- Age [Years]: 20-29 (23.8%), 30-39 (34.3%), 40-49 (29.1%), 50-59 (8.2%), 60-69 (1.4%)<br>-Sex [Males]: 84.3%<br>- Education: Grades 1-8 (3.7%), Grades 9-11 (7.5%), Grade 12/GED (46.3%), College 1-3 yrs	Self-reported head trauma:<br>- Any report of head injury/sxs (85.1%)<br>- LOC (35.8%), blow to head (47.8%),	- Intersection: Corrections, Parole, court<br><br>- 100% convicted of two DUI offenses	Intervention:<br>- 2-wk residential program & community-based after care under supervision of probation officer<br>- Classroom and counseling session to educate patients about alcohol and substance abuse, gain insight in addiction, teach avoidance strategies, and provide support for behavioral change	Barriers:<br>- NR<br><br>Facilitators<br>- NR<br><br>Gaps:<br>- NR

Study (Author, Year, Country)	Study Design & Objective	Study Sample[a]	TBI Status	Criminal Justice Status	Rehabilitation Intervention, Team, Outcome	TBI-Specific Facilitators, Barriers, Gaps
	the subtle cognitive impairments caused by mild brain injury and long -term alcohol consumption	(29.1%), College graduate (9.7%), Graduate school (3.7%)	concussion (36.6%)		- One session on raising awareness of TBI and interaction of alcohol and brain injury<br><br>Rehabilitation Team:<br>- NR<br><br>Outcome:<br>- N/A to rehabilitation intervention<br><br>Funding of Rehabilitation Program/Intervention:<br>- NR	
Glorney et al., 2018<br><br>United Kingdom	Observational Study (Mixed Methods)<br><br>To explore the efficacy and efficiency of a specialist brain injury Linkworker service at HMP Drake Hall, a closed training and resettlement prison for women in England.	N= 100 (Referred to the Brain Injury Linkworker Service)<br>- Age [Mean ± SD, Years]: 38 ± 10<br>- Gender [Women]: 100.0%<br>- Ethnicity: White British (80.0%), White (Gypsy or Irish Traveller) (5.0%), Mixed (White and Asian) (5.0%), Other (10.0%)<br>- Marital Status (N=71): Single (76.0%), Married (14.0%), Civil Partnership (10.0%), Widowed (1.0%)<br>- Occupation prior to custody (N=31): No work-related activity (84.0%), academic or vocational training (16.0%)	- Screening: BISI<br><br>- 89.0% w/ TBI<br>- Causes: domestic violence (45.0%), road traffic accident (21.0%), unprovoked attacks (15.0%), falls when sober (6.0%), falls when using drugs/alcohol (5.0%), sports injuries (4.0%), fights (3.0%)<br>- Age at time of injury [Mean ± SD, Years]: 25 ± 12	- Intersection: Corrections, parole<br><br>- Sentence length: <6 mos (0.9%), 6 to <12 mos (1.8%), 12 mos to < 2 yrs (9.6%), 2 yrs to < 4 yrs (28.5%), 4 yrs to < 10 yrs (42.8%), 10+ yrs (not life) (10.5%), indeterminate sentence (0.9%), life (5.0%)	Intervention:<br>- Linkworker service: Work w/people w/ brain injury to develop a pathway of support and optimize prisoner engagement w/sentence plan requirements and rehabilitation, and transition from custody to the community<br>- support w/psychoeducation, cognitive assessment and remediation, emotional management w/focus on brain injury, behavioural management, advocacy and health and well-being<br><br>Rehabilitation Team:<br>- Linkworker, consultant clinical psychologist<br><br>Outcome:<br>- Linkworker services improved self-esteem and confidence<br>- Improved use of practical techniques to assist w/memory<br>- Provided safeguarding for women in prison w/a brain injury; transitioning into a safe home<br><br>Funding of Rehabilitation Program/Intervention:<br>- NR	Barriers:<br>- Constraints on service resources meant that there were limits to the depth of training provided to prison staff, possibly leading to barriers to information sharing and limits to the Linkworker contributing to sentence planning<br>- Service lengths are long and women often lacked concentration<br><br>Facilitators:<br>- NR<br><br>Gaps<br>- Need for prison environment/ culture and staff to enable a holistic approach to supporting women w/TBI by complementing services of the Linkworker (i.e., training prison staff to avoid overreliance of women on Linkworkers)<br>- Need to integrate Linkworker service w/in broader prison

Study (Author, Year, Country)	Study Design & Objective	Study Sample[a]	TBI Status	Criminal Justice Status	Rehabilitation Intervention, Team, Outcome	TBI-Specific Facilitators, Barriers, Gaps
						context and use of trauma-informed approaches<br>- Need to create framework for action that incorporates race, culture, and class considerations and communicates how the service is gender-responsive
Kreutzer et al., 1991<br><br>United States	Before After (No Control)<br><br>To examine incidence of alcohol use, drug use, and criminal behaviour among persons w/TBI referred for supported employment services	N= 74<br>- Age [Mean ± SD, Years]: 30.9 ± 9.3<br>- Sex [Males]: 81.0%<br>- Marital Status: Single (53.0%), engaged (1.0%), married (20.0%), separated (12.0%), divorced (14.0%)<br>- Education: college graduates (10.0%), some college education (31.0%), HS graduates (32.0%), some HS education (19.0%), did not reach HS (7.0%)	- 100.0% hx of TBI<br>- LOC <24 hr (12%), LOC >24 hrs (84%), no LOC (4%)<br>- LOC [Mean ± SD, Days]: 50.6 ± 50.8<br>- Yrs post-injury [Mean ± SD]: 6.4 ± 6.0	- Intersection: Corrections<br><br>- 17.8% pre-injury arrest; 9.5% post-injury arrest	Intervention:<br>- University-based supported employment program & Neuropsychological exam<br><br>Rehabilitation Team:<br>- NR<br><br>Outcome:<br>- N/A to rehabilitation intervention<br><br>Funding of Rehabilitation Program/Intervention:<br>- NR	Barriers:<br>- Problems associated w/crime and substance abuse likely contribute to difficulties finding and maintaining employment<br><br>Facilitators:<br>- NR<br><br>Gaps:<br>- NR
Kreutzer et al., 1995<br><br>United States	Cohort Study<br><br>To examine alcohol use patterns, arrest hx, behavioural characteristics, and psychiatric tx hx	N= 327<br>- Age [Mean ± SD, Years]: 35.1 ± 12.9<br>- Sex [Males]: 65.0%<br>- Marital Status: Single (35.3%), steady relationship (3.6%), engaged (4.9%), living w/mate (1.3%), married (34.4.%), separated (8.0%), divorced (10.3%), widowed (2.2%)<br>- Education: 1-8 yrs (6.8%), some HS (19.8%), HS graduate (33.8%), some college (25.7%), college graduate (9.0%), postgraduate (4.1%)	- LOC [Mean ± SD, Days]: 15.7 ± 28.6<br>- Wks post-injury [Mean ± SD]: 97.0 ± 170.5	- Intersection: Corrections<br><br>- 19.5% pre-injury arrest, 14.6% subsequently convicted<br>- 7.2% post-injury arrest, 5.0% convicted<br>- 4% pre- and post-injury arrest<br>- Offences: DUI of alcohol, drug possession,	Intervention:<br>- NR (study identified psychiatric tx hx)<br>- Location: Outpatient rehabilitation medicine clinic<br><br>Rehabilitation Team:<br>- NR<br><br>Outcome:<br>- 37.3% of patients w/pre-injury hx of arrest received psychiatric or psychological tx before/after TBI<br>- 19.3% of patients w/negative criminal hx reported having received psychiatric/psychological tx	Barriers:<br>- NR<br><br>Facilitators:<br>- NR<br><br>Gaps:<br>- NR

Study (Author, Year, Country)	Study Design & Objective	Study Sample[a]	TBI Status	Criminal Justice Status	Rehabilitation Intervention, Team, Outcome	TBI-Specific Facilitators, Barriers, Gaps
				disorderly conduct, public drunkenness, assaults, theft, trespassing, grand larceny	- 36.0% of patients arrested post-injury and 31.0% of patients not arrested received psychiatric/psychological tx<br><br>Funding of Rehabilitation Program/Intervention:<br>- NR	
Luiselli et al., 2000<br><br>United States	Cross-sectional<br><br>To describe a survey conducted w/ children and adolescents w/severe TBI and attended a community-based program of education and neurorehabilitation	N= 69<br>- Age [Range, Years]: 11-22<br>- Gender [Males]: 79.7%	- 59.1% TBI due to athletic injury, battering, fall, motor vehicle accident, unspecified closed head injury (determined through chart review)	- Intersection: Policing, court<br><br>- 31.9% hx of violation (determined through chart review)<br>- 72.2% hx of committing 2-5 violations<br>- highest incidence occurred for theft, substance use, physical assault, alcohol use<br>- 50% of offenses occurred before enrollment in centre, 13.6% during enrolment, and 36.3% both before and during enrollment	Intervention:<br>- Community-based education and neurorehabilitation centre serving children and adolescents through a model of neurorehabilitation that combines educational, medical, nursing, family, residential-living, and psychological services<br><br>Rehabilitation Team:<br>- NR<br><br>Outcome:<br>- N/A to rehabilitation intervention<br><br>Funding of Rehabilitation Program/Intervention:<br>- NR	Barriers:<br>- NR<br><br>Facilitators:<br>- NR<br><br>Gaps:<br>- NR
Luong et al., 2021 Canada	RCT<br><br>To examine the effect of	Incarcerated (N= 220)<br>- Age [Mean ± SD]: 38.1 ± 9.9<br>- Sex [Males]: 83.2%	- 53.5% hx of TBI (self-report questionnaire)	- Intersection: Corrections	Intervention:<br>- At Home/Chez Soi Housing First: Immediate receipt of housing support and additional	Barriers:<br>- NR<br><br>Facilitators:

Study (Author, Year, Country)	Study Design & Objective	Study Sample[a]	TBI Status	Criminal Justice Status	Rehabilitation Intervention, Team, Outcome	TBI-Specific Facilitators, Barriers, Gaps
	HF intervention and health-related risk factors on incarceration among adults w/experiences of homelessness and mental illness	- Ethnic Background: White (39.1%), Black (34.6%), Other ethnic minority (26.4%)<br><br><u>Not-incarcerated (N= 288)</u><br>- Age [Mean ± SD]: 42.1 ± 12.7<br>- Sex [Males]: 59.7%<br>- Ethnic Background: White (33.7), Black (34.4), Other ethnic minority (31.9)<br><br><u>Homelessness:</u><br>- Absolutely homeless or precariously housed (100%)		- 43.3% ≥1 incarcerated episode b/t 2009 and 2014	services and resources without requisites of sobriety or tx adherence<br>- Randomly assigned to HF or TAU; those w/high needs received ACT and those w/moderate needs received ICM<br><br><u>Rehabilitation Team:</u><br>- NR<br><br><u>Outcome:</u><br>- HF intervention did not significantly reduce risk of incarceration<br><br><u>Funding of Rehabilitation Program/Intervention:</u><br>- NR	- NR<br><br><u>Gaps:</u><br>- Addition of specific criminogenic-based support services to the HF program may help reduce CJS involvement<br>- While the HF approach is effective in helping people exit homelessness, additional MHSU, and income supports may be needed to reduce justice system involvement
Manchester et al., 2007<br><br>United Kingdom	Case Study<br><br>To measure how aggression and bullying behaviour is reduced in three clients during the EQUIP program	<u>N= 3</u><br><br><u>Patient DL:</u><br>- Age: 19 at the time of study, 12 at the time of injury<br>- Sex: Male<br><br><u>Patient MG:</u><br>- Age: 21 at time of study, 19 at the time of TBI<br>- Sex: Male<br>- Sleeping on the streets prior to admission<br><br><u>Patient CD:</u><br>- 19 at the time of study, 17 at the time of TBI and criminal record began at 16 yrs old<br>- Sex: Male	- All three patients had severe TBI through motor vehicle traffic accidents<br>- GCS: DL (4), MG (3), CD (3)	- Intersection: Corrections, court<br><br>- Patient DL: Arrested and convicted for shoplifting<br>- Patient MG: no criminal hx<br>- Patient CD: criminal record from age of 16 yrs w/convictions for theft and criminal damage	<u>Intervention:</u><br>- EQUIP program – manual-based gp tx approach focused on teaching pro-social skills related to aggression, increasing moral developing, and altering pro-aggressive attitude<br>- Focuses on moral development, the development of anger management skills, and social skills training<br>- In the gp therapy, clients develop mutual helping skills<br><br><u>Rehabilitation Team:</u><br>- NR<br><br><u>Outcomes:</u><br>- Aggression and bullying behaviour reduced during EQUIP program and maintained at 3 mos follow-up<br>- Self-esteem measure did not alter across assessment period<br><br><u>Funding of Rehabilitation Program/Intervention:</u><br>- NR	<u>Barriers:</u><br>- NR<br><br><u>Facilitators:</u><br>- Motivational interviewing encourages initial attendance<br>- Factors that account for continued motivation include peer approval, staff encouragement, and high priority afforded<br><br><u>Gaps</u><br>- NR

Study (Author, Year, Country)	Study Design & Objective	Study Sample[a]	TBI Status	Criminal Justice Status	Rehabilitation Intervention, Team, Outcome	TBI-Specific Facilitators, Barriers, Gaps
McColl et al., 2010<br><br>Gaza, Egypt, Mexico, Honduras and South Africa	Cohort Study<br><br>To determine the feasibility of collecting data on demographics, torture exposure, and clinical needs from population of torture survivors attending rehabilitation centres for torture survivors and their recovery trajectories	N= 306<br>- Age [Mean ± SD, Years]: 37.7 ± 14.2<br>- Sex [Males]: 56.0%<br>- Religion: Christianity (55.0%), Islam (38.0%)<br>- Marital Status: Single (40.0%), Married/permanent relationship (42.0%), divorced/separated (10.0%), widowed (8.0%)<br>- Living setting: living at home (82.0%), prison (11.0%), camps/shelters (5.0%), homeless (2.0%)<br>- Education: primary education not completed (21.0%), primary education completed (26.0%), secondary education completed (30.0%), tertiary education qualification (10.0%), unknown (13.0%)<br>- Employment: 45% employed, 5% retired, 11% taking care of home and family, 7% studying, 32% unemployed/searching for work	- 61.4% beatings to the head	- Intersection: Corrections<br>- 11.0% living in prison	Intervention:<br>- Rehabilitation centres offered the following services: legal, employment, counselling/psychotherapy, social services, interpretation services, inpatient care, medical outpatient (adult), crisis/emergency response, educational services, medical outpatient (child and family), substance abuse, and alternative medical services<br><br>Rehabilitation Team:<br>- Service providers from medicine, psychology, nursing, and social work<br><br>Outcomes:<br>- Mean number of sxs and measures of depressive sxs dropped significantly over time (baseline to 3 mos to 6 mos)<br><br>Funding of Rehabilitation Program/Intervention:<br>- NR	Barriers:<br>- NR<br><br>Facilitators:<br>- NR<br><br>Gaps:<br>- NR
Miles et al., 2021<br><br>United States	Cohort Study<br><br>To examine rates and predictors of arrests in veterans and service members who received inpatient rehabilitation for TBI	N= 948<br>- Age at index TBI [median]: 30<br>- Sex [Males]: 94.0%<br>- Race/Ethnicity: White (67.0%), Black (9.0%), Hispanic (14.0%), Other (10.0%)<br>- Education: >HS diploma (57.0%), <HS diploma (43.0%)<br>- Marital status: Single (32.0%), Married (44.0%), Divorced (18.0%), separated (4.0%), widowed (1.0%))<br>- Annual earnings: <$50,000 (66.0%), ≥$50,000 (34.0%)	- 100.0% TBI dx<br>- Cause of injury: vehicular (47.0%), fall (15.0%), violence - penetrating (4.0%), other (34.0%)<br>- Severity: mild (20.0%), moderate (16.0%), severe (64.0%)	- Intersection: Corrections<br><br>- 34.0% arrested pre-injury; 14.0% penal incarcerations pre-injury<br>- 7.0% arrested post-TBI<br>- CJS involvement self-reported	Intervention:<br>- NR (participants were from TBI Model Systems Centres)<br>- Location: Comprehensive inpatient rehabilitation<br><br>Rehabilitation Team:<br>- NR<br><br>Outcomes:<br>- N/A to rehabilitation intervention<br><br>Funding of Rehabilitation Program/Intervention:	Barriers:<br>- NR<br><br>Facilitators:<br>- NR<br><br>Gaps:<br>- Need to assess for premorbid mental health sx and alcohol use in V/SM rehabilitation programs<br>- Need for appropriate environmental modifications at discharge (e.g., information

Study (Author, Year, Country)	Study Design & Objective	Study Sample[a]	TBI Status	Criminal Justice Status	Rehabilitation Intervention, Team, Outcome	TBI-Specific Facilitators, Barriers, Gaps
			- Median 5 yrs since index TBI		- Dept. of Veterans Affairs, Dept of Health & Human Services (National Institute on Disability, Independent Living, and Rehabilitation Research)	and transportation for Alcoholics Anonymous meetings)
Mitchell et al., 2021<br><br>New Zealand	RCT<br><br>To determine whether a psychological intervention improves coping, post-concussion sxs, and decreases in-prison infractions in adult males w/hx of TBI	N=55<br>- Age [Mean ± SD, Years]: 37.29 ± 9.81<br>- Sex [Males]: 100.0%<br>- Ethnicity: Māori (60.0%), European (15.0%), Pasifika (18.0%), Other (7.0%)	- 100.0% TBI (Self-reported)	- Intersection: Corrections<br><br>- Prison sentence length [Years]: 1-5 (38.0%), 5.1-10 (20.0%), 10.1-15 (13.0%), 15.1-20 (4.0%), 20+ (25.0%)<br>- Crimes: violence (47.0%), burglary (9.0%), sexual (27.0%), other (16.0%)	Intervention:<br>- Based on manualized 5-wk CBT/MBSR group-based program<br>- 10 sessions, in-person, group-based<br>- Sessions include development of personal goals, psycho-education, MBSR of thoughts and emotions, CBT, mindfulness exercises, integration of CBT, and multiple points of feedback<br><br>Rehabilitation Team:<br>- Intervention psychologist and intern psychologist, Prison mental health team<br><br>Outcomes:<br>- Improvement in use of calming and distraction strategies in intervention gp from baseline to post-intervention and 12 wk follow-up<br>- Participants in intervention gp had significantly higher negative affect repair on calming and distractive strategies subscale following completion of intervention, compared to wait-list controls<br>- Improvement in use of calming and distractive strategies not sustained at 12 wk follow-up<br><br>Funding of Rehabilitation Program/Intervention:<br>- NR	Barriers:<br>- High attrition rate due to participants being transferred or released<br><br>Facilitators:<br>- NR<br><br>Gaps:<br>- Explore the influence of readiness to change on retention and outcome<br>- Consider the location of the intervention delivery<br>- Exploring ways for participants to continue with the intervention post-release<br>- Need for procedures to allow for follow up of participants within the community
Nagele et al., 2019	Cohort Study<br><br>To describe demonstration project	N = 163<br>- Gender [Men]: 100%	- Screening: TBIQ<br><br>- 76.0% reported ≥1 event(s) that	- Intersection: Corrections, parole	Intervention:<br>- NeuroResource Facilitation – to identify resources and provide hands-on, ongoing support to individuals and families to access needed resources and services	Facilitators:<br>- NR<br><br>Barriers:<br>- NR

Study (Author, Year, Country)	Study Design & Objective	Study Sample[a]	TBI Status	Criminal Justice Status	Rehabilitation Intervention, Team, Outcome	TBI-Specific Facilitators, Barriers, Gaps
United States	w/men from maximum-security prison		could have resulted in TBI<br>- 89.0% mild TBI, of whom 34% experienced LOC, 66% being dazed/confuse<br>- 6.0% severe TBI<br>- 33% vehicular crashes, 30% assaults, 16% sports, 16% falls	- 100.0% (inmates in Transitional Housing Unit program at State Correctional Institution-Graterford, a maximum security prison	- Identify brain injury needs, assist people in applying for services needed<br>- Brain injury education and counselling, resource identification and application, advocacy, transportation training, medical case management and support, and the development of compensatory strategies<br>- Prior to release, focus on brain injury education, re-entry planning, and resource application<br>- Referral to state vocational rehab agency (where relevant)<br><br><u>Rehabilitation Team:</u><br>- NeuroResource Facilitator (Brain injury specialist w/20+ yrs of experience in working w/people w/ABI)<br>- Prison vocational staff<br>- Neuropsychologist/clinical neuropsychologist<br>- Prison employed psychological services specialist<br><br><u>Outcomes:</u><br>- 67 participants entered in intervention<br>- 61% assisted to apply for health insurance, 46% to apply for income benefit<br>- 61% assisted to apply for Office of Vocational Rehabilitation, 28% Pennsylvania Head Injury Program, 9% Home and Community Based Medicaid Waivers<br>- 46% of brain injury resources applied authorized at end of study<br>- 50% of released inmates went to Community Corrections setting/halfway house, 45% directly home<br><br><u>Funding of Rehabilitation Program/Intervention:</u>	<u>Gaps:</u><br>- Need for brain injury screening and assessment to be incorporated into routine health assessment in Corrections environments<br>- Need to train and educate corrections and parole personnel about brain injury<br>- Need to provide NeuroResource Facilitation before release and upon reentry<br>- Need to implement NeuroResource Facilitation as early as possible (e.g., with youth offenders)

Study (Author, Year, Country)	Study Design & Objective	Study Sample[a]	TBI Status	Criminal Justice Status	Rehabilitation Intervention, Team, Outcome	TBI-Specific Facilitators, Barriers, Gaps
					- Byrne Justice Assistance grant through the Pennsylvania Commission on Crime and Delinquency	
Pachalska et al., 2008<br><br>Poland	Case Study<br><br>To describe a 66- yr-old male patient w/long hx of schizophrenia, whose psychotic sxs displayed qualitative and quantitative changes after a closed-head injury	N= 1<br>- Age [Years]: 66<br>- Sex: Male<br>- Marital Status: Single<br>- Employment: Unemployed and on disability pension	- Hallucinating that he had wings and could fly, which prompted him to rush out into traffic to catch a bus, resulting in him struck by a passing automobile<br>- Suffered closed-head injury w/brain contusion<br>- LOC for 4–5 hrs	- Intersection: Corrections, court<br><br>- Incarceration for 6 mos and after this incident, arrested and sentenced for short prison terms	Intervention:<br>- Neuropsychological rehabilitation program: Goal-based program focused on working memory, perseveration, neglect, and executive functions<br>- Cognitive therapy, expanded art therapy<br><br>Rehabilitation Team:<br>- NR<br><br>Outcome:<br>- No trace of perseveration, either in his speech or in his drawings, and the slight hemispatial neglect has disappeared<br>- Some weakness in working memory, both visual and verbal, however, his IQ has improved noticeably, from borderline impaired to levels near normal (supported by MMSE results)<br>- Psychotropic drugs (haloperidol) gradually withdrawn and is now coping well without medication<br><br>Funding of Rehabilitation Program/Intervention:<br>- NR	Barriers:<br>- NR<br><br>Facilitators:<br>- NR<br><br>Gaps:<br>- NR
Pittaway et al., 2012<br><br>United Kingdom	Case Study<br><br>To describe a case of breach of duty, causation and contributory negligence	N= 1<br>- Age [Years]: 29<br>- Sex: Male<br>- Education: “Attended normal schools”<br>- Employment record: erratic and in unskilled jobs	- Major seizure caused patient to fall off a bunk bed<br>- GCS: 3	- Intersection: Corrections, parole, court<br><br>- B/t 1994 and 1997, three brief admissions to prison for theft	Intervention:<br>- NR (was transferred to neurorehabilitation centre)<br><br>Rehabilitation Team:<br>- NR<br><br>Outcome:<br>- N/A to rehabilitation intervention	Barriers:<br>- NR<br><br>Facilitators:<br>- NR<br><br>Gaps:<br>- NR

Study (Author, Year, Country)	Study Design & Objective	Study Sample[a]	TBI Status	Criminal Justice Status	Rehabilitation Intervention, Team, Outcome	TBI-Specific Facilitators, Barriers, Gaps
					Funding of Rehabilitation Program/Intervention:<br>- NR	
Ramos et al.,<br>2018<br><br>United Kingdom	Case Study<br><br>To develop, implement, and evaluate a brain injury Linkworker approach designed to support prisoners who report significant TBI/multiple mild TBIs	N= 3<br><br>VW:<br>- Age [Years]: 22<br>- Sex: Male<br>- Education: Attended college<br>- Employment: Unemployed<br><br>RR<br>- Age [Years]: 47<br>- Sex: Male<br>- Living environment: Homeless/living in hostel (after separation from partner)<br><br>MB<br>- Age [Years]: 40<br>- Sex: Male	- Screening: BISI<br><br>- VW: TBI as a result of assault<br>- RR: Self-reported head injuries that resulted in LOC<br>- MB: TBI as a result of road traffic incident	- Intersection: Corrections<br><br>- VW: Forensic hx prior to TBI and incidents of criminal damage<br>- RR: Stealing cars<br>- MB: Assault on wife, premorbid hx of aggression, and served prison sentence grievous bodily harm	Intervention:<br>- The Brain Injury Linkworker Service – to offer direct help to prisoners referred to them by providing psychoeducation concerning brain injury and by helping individuals to develop strategies to circumvent brain<br>- Phase I – Identifying hx and consequences of TBI<br>- Phase II – Intervention in custody, tailored to individual needs, person-centred<br>- Phase III – Follow-up after release<br><br>Rehabilitation Team:<br>- Linkworkers (psychology graduates, clinical psychologist)<br><br>Outcome:<br>- VW: After 6 mos of intensive support, able to live independently w/little support from PT, SW, Disabilities Trust Community Services, probation officer, and family<br>- RR: He gained a job as an “advisor” w/prison-based charity<br>- MB: positive outcomes while in custody; Linkworker secured a place at neurobehavioural rehabilitation, but his prison sentence ended without court order for him to receive rehabilitation; no follow-up information available<br><br>Funding of Rehabilitation Program/Intervention:<br>- NR	Barriers:<br>- Difficulty differentiating problems arising from TBI and from other causes, as problems (e.g., mental health disorders) overlap<br><br>Facilitators:<br>- Intervention was deliberately designed to be low cost<br><br>Gaps:<br>- NR
Sander et al.,<br>2018	Cohort Study	N= 7685<br><br>White (N=5,548)	- 100.0% TBI (documented using the	- Intersection: Corrections	Intervention:<br>- NR (participants were from TBI Model Systems Centres)	Barriers:<br>- NR

Study (Author, Year, Country)	Study Design & Objective	Study Sample[a]	TBI Status	Criminal Justice Status	Rehabilitation Intervention, Team, Outcome	TBI-Specific Facilitators, Barriers, Gaps
United States	To investigate contribution of race/ethnicity to retention in TBI research at 1 to 2 yrs post-injury	- Age [Mean ± SD, Years]: 42.8 ± 20.0<br>- Sex [Males]: 71.9%<br>- Pre-injury marital status: Married (37.2%), not married (62.8%)<br>- Pre-injury education: ≤8th grade (3.4%), 9th to 11th grades (15.3%), 12th grade (HS/GED) (37.7%), >12th grade (43.6%)<br><br>Black (N=1,347)<br>- Age [Mean ± SD, Years]: 38.6 ± 17.0<br>- Sex [Males]: 77.9%<br>- Pre-injury marital status: Married (19.9%), Not married (80.1%)<br>- Pre-injury education: ≤8th grade (6.3%), 9th to 11th grades (27.4%), 12th grade (HS/GED) (39.1%), >12th grade (27.3%)<br><br>Hispanic (N=790)<br>- Age [Mean ± SD, Years]: 35.9 ± 17.6<br>- Sex [Males]: 77.6%<br>- Pre-injury marital status: Married (27.5%), Not married (72.5%)<br>- Pre-injury education: ≤8th grade (25.0%), 9th to 11th grades (25.4%), 12th grade (HS/GED) (27.6%), >12th grade (22.1%)	emergency department GCS)<br><br>Cause of injury:<br>- White: 6.1% violent, 93.9% non-violent<br>- Black: 23.6% violent, 76.4% non-violent<br>- Hispanic: 17.3% violent, 82.7% non-violent	Pre-injury penal incarcerations:<br>- White: 7.1%<br>- Black: 19.0%<br>- Hispanic: 10.4%	- Location: Comprehensive inpatient rehabilitation<br><br>Rehabilitation Team:<br>- NR<br><br>Outcome:<br>- N/A to rehabilitation intervention<br><br>Funding of Rehabilitation Program/Intervention:<br>- NR	Facilitators<br>- NR<br><br>Gaps:<br>- NR
Simpson et al., 1999<br><br>Australia	Cohort Study<br><br>To describe the nature and extent of	N =29 w/TBI<br>- Age [Mean ± SD, Years]: 32 yrs ± 10.0<br>- Gender [Males]: 100.0%	- Age at time of injury [Mean ± SD, Years]: 22 ± 10.2	- Intersection: Corrections, Parole	Intervention:<br>- NR (participants were from the Brain Injury Rehabilitation Unit)<br><br>Rehabilitation Team:	Barriers:<br>- NR<br><br>Facilitators:<br>- NR

Study (Author, Year, Country)	Study Design & Objective	Study Sample[a]	TBI Status	Criminal Justice Status	Rehabilitation Intervention, Team, Outcome	TBI-Specific Facilitators, Barriers, Gaps
	sexual offending after TBI	Pre-injury:<br>- Relationship status: married/de facto (20.0%), separated/divorced (15.0%), single (65.0%)<br>- Vocational status: professional/managerial/skilled (30.0%), unskilled/semi-skilled (30.0%), student (25.0%), other (15.0%)<br><br>Post-injury:<br>- Relationship status: married/de facto (5.0%), separated/divorced (30.0%), single (65.0%)<br>- Vocational status: professional/managerial/skilled (3.0%), in rehabilitation (30.0%), avocational (64.0%), other (3.0%)	- Time post-injury [Mean ± SD, Years]: 10 ± 7.1<br>- Retrospective case file review	- Sexual offenses: exhibitionism (w/ or without masturbation), frotteurism, toucherism, voyeurism, overt sexual aggression, other	- NR<br><br>Rehabilitation Outcome:<br>- N/A to rehabilitation intervention<br><br>Funding of Rehabilitation Program/Intervention:<br>- State-funded	Gaps:<br>- Need for specialized training programs to enhance staff security, as staff were the most common target of offenses<br>- Need to examine instances of non-consensual non-genital touching as possible sexual offences as opportunities to teach appropriate social skills<br>- Need for close assessment of behaviour in family and service environments
Tate et al., 1998<br><br>Australia	Cohort Study<br><br>To examine influence of premorbid psychosocial variables in determining rehabilitation outcomes after TBI	Study 1:<br><br>No pre-morbid social maladjustment (N=11):<br>- Yrs of schooling [Mean ± SD]: 9.9 ± 1.3<br>- Occupation: professional/ managerial (9.0%), clerical/ sales (9.0%), skilled (27.0%), semiskilled (45.0%), unskilled (9.0%),<br><br>Pre-morbid Social Maladjustment (N= 11):<br>- Yrs of schooling [Mean ± SD]: 8.9 ± 1.4<br>- Gender [Males]: 100.0%<br>- Occupation: clerical/sales (9.0%), semiskilled (36.0%),	Study 1:<br><br>No pre-morbid social maladjustment (N=11):<br>- Age at injury [Mean ± SD]: 26.8 ± 8.2<br><br>Pre-morbid social maladjustment (N= 11):<br>- Age at injury [Mean ± SD]: 26.8 ± 9.4<br><br>Study 2:<br>Poor Histories	- Intersection: Corrections<br><br>Study 1:<br>- Criminal record and previous gaol sentence (N=4), hx of delinquent behaviour resulting in noncustodial sentences (N=3), hx of substance abuse (N=3), criminal record and hx of barbiturate abuse (N=1)	Intervention:<br>- NR (participants were from regional brain injury rehabilitation unit)<br><br>Rehabilitation Team:<br>- NR<br><br>Outcomes:<br>- N/A to rehabilitation intervention<br><br>Funding of Rehabilitation Program/Intervention:<br>- NR	Barriers:<br>- NR<br><br>Facilitators:<br>- NR<br><br>Gaps:<br>- NR

Study (Author, Year, Country)	Study Design & Objective	Study Sample[a]	TBI Status	Criminal Justice Status	Rehabilitation Intervention, Team, Outcome	TBI-Specific Facilitators, Barriers, Gaps
		unskilled (27.0%), unemployed (27.0%)<br><br>Study 2: N= 30<br>Poor Histories<br>- Yrs of schooling [Mean ± SD]: 9.7 ± 1.4<br><br>Good Histories<br>- Yrs of schooling [Mean ± SD]: 10.6 ± 1.2	- Age at injury [Mean ± SD]: 25.0 ± 9.6<br><br>Good Histories<br>- Age at injury [Mean ± SD]: 26.6 ± 8.5	Study 2:<br>- criminal delinquency record (N=6)		
Topolovec-Vranic et al., 2017<br><br>Canada	RCT<br><br>To characterize prevalence of self-reported head injury w/ LOC and associated demographic, clinical, and service use factors in a sample of homeless adults w/ mental illness	N= 2,088<br><br>No hx of head injury + head injury w/out LOC (N=990)<br>- Age [Mean ± SD, Years]: 41.0 ± 11.9<br>- Sex [Males]: 63.4%<br>- Sex [Other]: 1.0%<br>- Education: <HS (51.3%), completed HS (20.2%), some college/university (28.5%)<br>- Ethnicity: Aboriginal (13.5%), Ethno-racial (31.2%), White (55.2%)<br>- Housing Status: Absolutely homeless (84.9%), precariously housed (15.1%)<br><br>Head Injury w/LOC (N= 1,098)<br>- Age [Mean ± SD, Years]: 40.8 ± 10.6<br>- Sex [Males]: 71.4%<br>- Sex [Other]: 0.8%<br>- Education: <HS (59.0%), completed HS(18.1%), some college/university (22.9%)<br>- Ethnicity: Aboriginal (28.9%), Ethno-racial (18.8%), White (52.4%)	- 52.6% hx of TBI (self-reported)<br>- Median (IQR) number of TBIs: 3 (1, 6)<br>- Median (IQR) of LOC (hrs): 1.0 (0.10, 8.0)	- Intersection: Corrections, parole<br><br>- Contact w/CJS in past 6 mos: 30.7% (No hx of head injury + head injury w/out LOC), 40.0% (head injury w/LOC)	Intervention:<br>- At Home/Chez Soi Housing First: Immediate receipt of housing support and additional services and resources without requisites of sobriety or tx adherence<br>- Randomly assigned to HF or TAU; those w/high needs received ACT and those w/moderate needs received ICM<br><br>Rehabilitation Team:<br>- NR<br><br>Outcome:<br>- HF intervention did not significantly reduce risk of incarceration<br><br>Funding of Rehabilitation Program/Intervention:<br>- NR	Barriers:<br>- NR<br><br>Facilitators:<br>- NR<br><br>Gaps:<br>- Need to consider addition of appropriate supportive care and living arrangements alongside tx plans

Study (Author, Year, Country)	Study Design & Objective	Study Sample[a]	TBI Status	Criminal Justice Status	Rehabilitation Intervention, Team, Outcome	TBI-Specific Facilitators, Barriers, Gaps
		- Housing Status: Absolutely homeless (78.6%), precariously housed (21.4%)				
Williams & Chitsabesan, 2013-2015<br><br>England	Cohort Study<br><br>To evaluate whether the Linkworker service is designed in accordance w/best practice evidence, and meeting its target of the identification and management of TBI in young people who offend	N= 65 (14 provided consent to share demographic information):<br>- Age [Years]: 15-18 (N=7), 18-22 (N=7)<br>- Sex [Males]: 100.0%<br>- Ethnicity: White (65.0%), Asian (21.0%), Not recorded (14.0%)<br>- Pre-custody living arrangements: immediate family (63.0%), children's home (21.0%), friends (7.0%), other (7.0%)<br>- School/college or work prior to custody: Not in education, employment, or training (49.0%), vocational training (14.0%), not known (14.0%), work (14.0%), school/college (7.0%)<br><br>Service Users at Male Young Person Center (N=32):<br>- Age [Years]: 16-18<br>- Sex [Males]: 100.0%<br><br>Service Users at Young Offender Institutions and Adult Male Category C (N=33):<br>- Age [Years]: 15-18 (10), 18-22 (23)<br>- Sex [Males]: 100.0%	- Screening: CHAT<br><br>N= 65; 14 provided consent<br>- Age at injury [Mean, Years]: 9.8<br>- Severity: mild (21.0%), moderate to severe (71.0%)<br>- Causes: fall when sober (N=5), fall under the influence (N=3), road traffic accidents (N=6), assaults (N=6), fights (N=10), work (N=1)<br><br>Service Users at Male Young Person Center (N=32):<br>- Age at injury [Mean, Years]: 10.9 (N=24)<br>- Severity: mild (34.0%), moderate to severe (66.0%)<br>- Causes: fall when sober (N=10), fall	- Intersection: Corrections<br><br>- 100.0% CJS<br>- Frequency of previous custody: Once (42.0%), twice (21.0%), 3 times (14.0%), 4 times (14.0%), 5 times (7.0%)<br>- Current Offence: Non-violent (49.0%), violent (35.0%), sexual (7.0%), multiple (7.0%)	Intervention:<br>- Linkworker: one-to-one support; education about brain injury and its effects, cognitive strategies involving functional intervention aids, behavioural management plans and guidelines<br><br>Rehabilitation Team:<br>- Linkworkers working w/ education personnel, mental health nurses, keyworker, youth offending team<br><br>Outcome:<br>- service improved management of mood, remembering, stopping to think, to be less aggressive<br>- intervention was helpful for getting a job, avoiding fights, getting into less trouble, maintaining better relationships<br><br>Funding of Rehabilitation Program/Intervention:<br>- Disabilities Trust Foundation and Barrow Cadbury Trust	Barriers:<br>- Difficulty in accessing prison training and establishing the service<br>- Linkworkers fitting in with the prison regime as the service is small, and there is often lack of communication and missed appointments<br>- Families and clients preferred to have home visits<br>- Clients having to complete so much paperwork<br><br>Facilitators:<br>- Multidisciplinary meetings increased connections with other services<br><br>Gaps:<br>- NR

Study (Author, Year, Country)	Study Design & Objective	Study Sample[a]	TBI Status	Criminal Justice Status	Rehabilitation Intervention, Team, Outcome	TBI-Specific Facilitators, Barriers, Gaps
			when under the influence (N=4), road traffic accident (N=7), fight (N=7), sports injury (N=6), other (N=24)<br><br>Service Users at Young Offender Institutions and Adult Male Category C (N=33):<br>- Age at injury [Mean, Years]: 10.5<br>- Severity: mild (30.0%), moderate to severe (70.0%)<br>- Causes: fall when sober (N=16), fall when under the influence (N=14), road traffic accident (N=13), assault (N=10), other (N=5)			
Ylvisaker et al., 2007<br><br>United States	Cohort Study<br><br>To describe outcomes long-term outcome of a sample of individuals served by	Study 1: N= 51<br>- Sex [Males]: 74.5%	- TBI + substance abuse (N=19), TBI + mental health dx (N=27), TBI + substance abuse and mental health dx (N=5)	- Intersection: Corrections<br>- In correctional facilities prior to admission to community support program (N=9)	Intervention:<br>- Neurobehavioural Resource Project – facilitate successful sustained community living by providing effective behavioural services, both for individuals w/TBI and for those who support them in the community<br>- Offers direct services to individuals w/severe behavioural difficulties, staff training, including an apprenticeship program for staff	Barriers:<br>- NR<br><br>Facilitators:<br>- NR<br><br>Gaps:

Study (Author, Year, Country)	Study Design & Objective	Study Sample[a]	TBI Status	Criminal Justice Status	Rehabilitation Intervention, Team, Outcome	TBI-Specific Facilitators, Barriers, Gaps
	New York State Dept of Health TBI Medicaid Waiver Program				throughout the state, and quality assurance monitoring<br><br>Rehabilitation Team:<br>- NR<br><br>Outcomes:<br>- 63.3% reported satisfaction w/employment/daily activity<br>- 76.7% reported choice in employment/daily activity<br>- 62.2% reported having transportation when needed<br>- 73.0% reported opportunity for community involvement<br><br>Funding of Rehabilitation Program/Intervention:<br>- NR	- Participant dissatisfaction (e.g., lack of transportation, limited opportunities for community involvement)

Notes:

[a] Study sample's characteristics are reported as described in the manuscript; for example, if the study used the term "gender" but described their participants as "males" or "females", this was reported as "Gender [Males]" in the table

ACT: Assertive Community Treatment; **BISI:** Brain Injury Screening Index; **B/t:** Between; **CBT:** Cognitive behavioural therapy; **CHAT:** Comprehensive Health Assessment Tool; **CI:** Confidence Interval; **CJS:** Criminal Justice Status; **d/c:** discharge; **Dept:** Department; **DUI:** Driving under the influence**; dx:** diagnosis; **GED:** General Educational Development; **GP:** General Practitioner; **GCS:** Glasgow Coma Scale; **GHHQ:** The General Health and History Questionnaire; **HF:** Housing First; **HR:** Hazard Ratio; **HS:** High school; **Hx:** History; **ICM:** Intensive Case Management; **MBSR:** Mindfulness-Based Stress Reduction; **IQR:** Interquartile Range; **MHSU:** Mental Health and/or Substance Use; **MMSE:** Mini-Mental State Exam; **Mos:** Months; **N/A:** Not Applicable; **NR:** Not reported; **LOC:** Loss of Consciousness; **OSU TBI-ID:** Ohio State University Traumatic Brain Injury Identification Method; **PT:** Physiotherapist; **RCT:** Randomized Control Trial; **SD:** Standard Deviation; **SWs:** Social Workers; **Sxs:** Symptoms; **TAU:** Treatment As Usual; **TBI:** Traumatic Brain Injury; **TBIQ:** Traumatic Brain Injury Questionnaire; **TBIMS NDB:** Traumatic Brain Injury Model Systems National Database; **Tx:** Treatment; **V/SM:** Veterans and Service Members; **w/:** with; **wks:** weeks; **Yr(s):** Year(s).
