# Supplementary material for: Rehabilitation among individuals with traumatic brain injury who intersect with the criminal justice system: A scoping review
Source: Front Neurol. 2023 Jan 17;13:1052294. doi: 10.3389/fneur.2022.1052294 (PMC9886883; doi:10.3389/fneur.2022.1052294)
Supplement: Supplementary file 3 [file Data_Sheet_3.pdf]

**Quality Appraisal for Controlled Interventions (N=3)**<br><br>**Authors**<br>**Year**<br>**Title**	Luong et al.<br>2021<br>The impact of a Housing First intervention and health-related risk factors on incarceration among people with experiences of homelessness and mental illness in Canada	Mitchell et al.<br>2021<br>An intervention to improve strategies in adult male prisoners with a history of traumatic brain injury: A pilot randomized clinical trial	Topolovec-Vranic et al.<br>2017<br>The high burden of traumatic brain injury and comorbidities amongst homelessness adults with mental illness
**CRITERIA**			
1. Was the study described as randomized, a randomized trial, a randomized clinical trial, or an RCT?	Yes	Yes	Yes
2. Was the method of randomization adequate (i.e., use of randomly generated assignment)?	NR	No	No
3. Was the treatment allocation concealed (so that assignments could not be predicted)?	NR	Yes	No
4. Were study participants and providers blinded to treatment group assignment?	NR	Yes	No
5. Were the people assessing the outcomes blinded to the participants' group assignments?	NR	NR	No
6. Were the groups similar at baseline on important characteristics that could affect outcomes (e.g., demographics, risk factors, co-morbid conditions)?	No	Yes	No
7. Was the overall drop-out rate from the study at endpoint 20% or lower of the number allocated to treatment?	NR	No	NR
8. Was the differential drop-out rate (between treatment groups) at endpoint 15 percentage points or lower?	NR	Yes	NR
9. Was there high adherence to the intervention protocols for each treatment group?	NR	NR	NR
10. Were other interventions avoided or similar in the groups (e.g., similar background treatments)?	Yes	CD	Yes
11. Were outcomes assessed using valid and reliable measures, implemented consistently across all study participants?	Yes	Yes	Yes
12. Did the authors report that the sample size was sufficiently large to be able to detect a difference in the main outcome between groups with at least 80% power?	No	Yes	NR
13. Were outcomes reported or subgroups analyzed prespecified (i.e., identified before analyses were conducted)?	Yes	Yes	Yes
14. Were all randomized participants analyzed in the group to which they were originally assigned, i.e., did they use an intention-to-treat analysis?	NR	Yes	NR
**Response Options:** Yes, No, CD (Cannot Determine), NR (Not Reported), or NA (Not Applicable)			

**Quality Appraisal for Observational Cohort Study or Cross-Sectional Studies (N=13)**<br>**Authors**<br>**Year**<br>**Title**	Bannon et al.<br>2020<br>Reinjury after moderate to severe TBI: Rates and risk factors in the NIDILRR traumatic brain injury model systems	Chitsabesan et al.<br>2015<br>Traumatic brain injury in juvenile offenders: Findings from the comprehensive health assessment tool study and the development of a specialist linkworker service	Dillahunt-Aspillaga et al.<br>2015<br>Predictors of behavioural health service use and associated expenditures: Individuals with TBI in Pinellas County	Glass et al.<br>2000<br>Cognitive impairment screening in second offense DUI programs	Kreutzer et al.<br>1995<br>Interrelationships between crime, substance abuse, and aggressive behaviours among persons with traumatic brain injury	Luisellie et al.<br>2000<br>Incidence of law-violating behaviour in a community sample of children and adolescents with traumatic brain injury	McColl et al.<br>2010<br>Rehabilitation of torture survivors in five countries: Common themes and challenges	Miles et al.<br>2021<br>Demographic and mental health predictors of arrests up to 10 years post-traumatic brain injury: A Veterans Affairs TBI Model Systems Study	Nagele et al.<br>2019<br>Brain injury in an offending population: Implications for re-entry and community transition	Sander et al.<br>2018<br>Race/ethnicity in retention in traumatic brain injury outcomes research: A Traumatic Brain Injury Model Systems National Database Study	Simpson et al.<br>1999<br>Sex offending as a psychosocial sequelae of traumatic brain injury	Tate, RL. (Study 1)<br>1998<br>"It is not only the kind of injury that matters, but the kind of head": The contribution of premorbid psychosocial factors to rehabilitation outcomes after severe traumatic brain injury	Tate, RL. (Study 2)<br>1998<br>"It is not only the kind of injury that matters, but the kind of head": The contribution of premorbid psychosocial factors to rehabilitation outcomes after severe traumatic brain injury	Ylvisaker et al.<br>2007<br>Long-term community supports for individuals who co-occurring disabilities after traumatic brain injury: Cost effectiveness and project-based intervention
**CRITERIA**														
1. Was the research question or objective in this paper clearly stated?	Yes	Yes	Yes	Yes	Yes	Yes	Yes	Yes	Yes	Yes	Yes	Yes	Yes	Yes
2. Was the study population clearly specified and defined?	Yes	Yes	Yes	Yes	Yes	Yes	Yes	Yes	Yes	Yes	Yes	Yes	Yes	Yes
3. Was the participation rate of eligible persons at least 50%?	Yes	No	Yes	Yes	NA	NA	No	NR	NR	NR	NR	NR	NR	Yes
4. Were all the subjects selected or recruited from the same or similar populations (including the same time period)? Were inclusion and exclusion criteria for being in the study prespecified and applied uniformly to all participants?	Yes	Yes	Yes	Yes	Yes	Yes	No	Yes	Yes	Yes	Yes	Yes	Yes	Yes
5. Was a sample size justification, power description, or variance and effect estimates provided?	No	No	No	No	NA	NA	No	No	No	No	No	No	No	No
6. For the analyses in this paper, were the exposure(s) of interest measured prior to the outcome(s) being measured?	Yes	Yes	NA	NA	NA	NA	Yes	Yes	Yes	Yes	Yes	Yes	Yes	Yes
7. Was the timeframe sufficient so that one could reasonably expect to see an association between exposure and outcome if it existed?	CD	NA	NA	NA	NA	NA	NR	CD	CD	NA	CD	CD	CD	NA
8. For exposures that can vary in amount or level, did the study examine different levels of the exposure as related to the outcome (e.g., categories of exposure, or exposure measured as continuous variable)?	Yes	NA	NA	NA	NA	NA	Yes	NA	Yes	NA	NA	NA	NA	NA
9. Were the exposure measures (independent variables) clearly defined, valid, reliable, and implemented consistently across all study participants?	Yes	NA	NA	NA	NA	NA	Yes	Yes	Yes	Yes	Yes	Yes	Yes	Yes
10. Was the exposure(s) assessed more than once over time?	NA	NA	NA	NA	NA	NA	Yes	Yes	NA	NA	NA	NA	NA	NA
11. Were the outcome measures (dependent variables) clearly defined, valid, reliable, and implemented consistently across all study participants?	Yes	NA	Yes	Yes	Yes	NA	Yes	Yes	Yes	Yes	Yes	Yes	Yes	Yes
12. Were the outcome assessors blinded to the exposure status of participants?	NA	NA	NA	NA	NA	NA	No	No	No	No	No	No	No	No
13. Was loss to follow-up after baseline 20% or less?	NA	NA	Yes	NA	NA	NA	No	No	NR	Yes	NR	NR	NR	NR
14. Were key potential confounding variables measured and adjusted statistically for their impact on the relationship between exposure(s) and outcome(s)?	Yes	NA	NA	NA	NA	NA	NA	Yes	NA	Yes	NA	NA	NA	NA
**Response Options:** Yes, No, CD (Cannot Determine), NR (Not Reported), or NA (Not Applicable)														

**Quality Appraisal for Before-After (Pre-Post) Studies with no Control Grp (N=1)**	
**Authors**	Kreutzer et al.
**Year**	1991
**Title**	Substance abuse and crime patterns among persons with traumatic brain injury referred for supported employment
**CRITERIA**	
1. Was the study question or objective clearly stated?	Yes
2. Were eligibility/selection criteria for the study population prespecified and clearly described?	Yes
3. Were the participants in the study representative of those who would be eligible for the test/service/intervention in the general or clinical population of interest?	Yes
4. Were all eligible participants that met the prespecified entry criteria enrolled?	CD
5. Was the sample size sufficiently large to provide confidence in the findings?	NR
6. Was the test/service/intervention clearly described and delivered consistently across the study population?	Yes
7. Were the outcome measures prespecified, clearly defined, valid, reliable, and assessed consistently across all study participants?	Yes
8. Were the people assessing the outcomes blinded to the participants' exposures/interventions?	NR
9. Was the loss to follow-up after baseline 20% or less? Were those lost to follow-up accounted for in the analysis?	NR
10. Did the statistical methods examine changes in outcome measures from before to after the intervention? Were statistical tests done that provided p values for the pre-to-post changes?	Yes
11. Were outcome measures of interest taken multiple times before the intervention and multiple times after the intervention (i.e., did they use an interrupted time-series design)?	No
12. If the intervention was conducted at a group level (e.g., a whole hospital, a community, etc.) did the statistical analysis take into account the use of individual-level data to determine effects at the group level?	Yes
**Response Options:** Yes, No, CD (Cannot Determine), NR (Not Reported), or NA (Not Applicable)	

Quality Appraisal for Case Studies/Case Series Studies (N=5)					
**Authors**	Bezeau et al.	Manchester et al.	Pachalska et al.	Pittaway et al.	Ramos et al.
**Year**	2004	2007	2008	2012	2018
**Title**	Sexually intrusive behaviour following brain injury: Approaches to assessment and rehabilitation	A forensic peer group approach to bullying after traumatic brain injury	Neuropsychological diagnosis and treatment after closed-head injury in a patient with a psychiatric history of schizophrenia	Prison medical care and status epilepticus	Brain injury and offending: The development and field testing of a Linkworker Intervention
**CRITERIA**					
1. Was the study question or objective clearly stated?	Yes	Yes	Yes	Yes	Yes
2. Was the study population clearly and fully described, including a case definition?	Yes	Yes	Yes	Yes	Yes
3. Were the cases consecutive?	NA	CD	NA	NA	CD
4. Were the subjects comparable?	NA	CD	NA	NA	CD
5. Was the intervention clearly described?	Yes	Yes	Yes	Yes	Yes
6. Were the outcome measures clearly defined, valid, reliable, and implemented consistently across all study participants?	Yes	Yes	Yes	No	Yes
7. Was the length of follow-up adequate?	NA	CD	NA	NA	CD
8. Were the statistical methods well-described?	NA	No	NA	NA	NA
9. Were the results well-described?	Yes	Yes	Yes	Yes	Yes
**Response Options:** Yes, No, CD (Cannot Determine), NR (Not Reported), or NA (Not Applicable)					
